# Functional Antigen‐Specific CD8 T_SCM_ Responses Are Associated with Repeated Clearance of Hepatitis C Virus Infection

**DOI:** 10.1002/eji.70098

**Published:** 2025-12-18

**Authors:** Yanran Zhao, Elizabeth Keoshkerian, Hui Li, Rachel Sacks‐David, Irene Boo, Paul Dietze, Margaret Hellard, Heidi Drummer, Fabio Luciani, Rowena A. Bull, Andrew R. Lloyd

**Affiliations:** ^1^ Viral Immunology Systems Program (VISP) The Kirby Institute University of New South Wales Sydney Australia; ^2^ School of Biomedical Sciences Faculty of Medicine University of New South Wales Sydney Australia; ^3^ Burnet Institute Melbourne VIC Australia; ^4^ Department of Microbiology Monash University Clayton VIC Australia; ^5^ Department of Microbiology and Immunology Peter Doherty Institute for Infection and Immunity University of Melbourne Melbourne VIC Australia

**Keywords:** CD8 T cell, HCV re‐infection, long‐lived protective immunity, memory T cell response, stem cell

## Abstract

Natural clearance of hepatitis C virus (HCV) infection occurs in about 25% of primary infections, but offers only partial protective immunity against re‐infections. This study hypothesised that long‐lived polyfunctional HCV‐specific CD8+ memory stem T cells (T_SCM_) contribute to protective immunity in rare super‐clearer subjects who repeatedly clear viraemia. Six super‐clearers and four clearer‐chronic subjects who resolved a primary infection but subsequently developed chronic infection were studied at multiple timepoints. The T_SCM_ population (CCR7+CD45RA+CD95+) was bulk sorted, labelled with CellTrace Violet (CTV), and stimulated in vitro for five days with cognate HCV peptide, IL‐2/IL‐15, and autologous PBMCs. Functionality of the expanded HCV‐specific T_SCM_ was assessed via the proliferation, multi‐potency, and stemness indices. Total HCV‐specific CD8+ T cells from super‐clearers exhibited enhanced proliferative recall capability compared with clearer‐chronics. Furthermore, super‐clearers exhibited higher HCV‐T_SCM_ frequencies post‐expansion (22.35 ± 34.35 vs. 2.41 ± 9.83; *p* = 0.0066). Notably, HCV‐T_SCM_ in clearer‐chronics had ‘stemness’ indices of zero in samples before the re‐infection (i.e., no ability to generate T_SCM_ as progeny), whereas super‐clearers consistently retained this key functional property. These findings suggest that the maintenance of self‐renewing HCV‐specific T_SCM_ may underpin long‐term protective immunity against re‐infection and could inform vaccine design strategies targeting durable cellular memory.

## Graphical abstract

HCV re‐infection is common after spontaneous clearance of the initial infection. In the rare group of super‐clearers who clear repeatedly, HCV‐specific CD8+ T memory stem cells (T_SCM_) retain stemness, proliferation, and multipotency, sustaining immune protection. In contrast, clearer chronics lose these properties. T_SCM_ self‐renewal underpins durable immunity and should inform vaccine design.

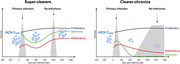

## 1 Introduction

Natural clearance of hepatitis C virus (HCV) occurs in approximately 25% of cases after primary infection, but confers only partially protective immunity against subsequent infection, with the risk of developing chronic infection upon re‐exposure recognised to be reduced, but not completely so, compared with the primary infection outcomes [1, 2, 3, 4]. It has been suggested that HCV‐specific memory CD8 T cell responses that are established after primary infection contribute to protection against the establishment of chronic viraemia upon re‐infection [1, 5, 6]. In chimpanzees that were subjected to a series of HCV re‐infection experiments [6], significant frequencies of HCV‐specific CD8 T cells recognising multiple epitopes were evident in the peripheral blood one month after primary infection and remained stable over 7 years of follow‐up. Re‐challenge with identical viral strains after 7 years induced rapid expansion of long‐lived antigen‐specific memory T cells that produced IFN‐γ and upregulated the activation marker, CD69. HCV viraemia was terminated within 14 days in those animals, in contrast to the prolonged course of the first infection [6]. Another key study in humans also showed that efficient control of secondary infection was temporally linked to the rapid acquisition of effector functions and expansion of HCV‐specific memory CD8 T cells. HCV re‐infection was associated with a greater magnitude and breadth of HCV‐specific T cells in the five subjects who cleared the re‐infection in comparison to four subjects who became chronically infected. The HCV‐specific CD8 T cells in the subjects who successfully cleared repeated infections demonstrated greater poly‐functionality (i.e., producing more than one cytokine) and expansion of CD127^lo^ HCV‐specific memory CD8 T cells after virus re‐challenge, suggesting a pre‐existing subset of memory T cells responded rapidly to the second virus with differentiation into CD127^lo^ effector T cells [5].

Homeostatic maintenance of the CD8 memory T cell pool is recognised to rely on IL‐7 and IL‐15 [7]. In relation to HCV infection, expression of CD127 (the alpha chain of the IL‐7 receptor) on CD8 T cells is usually considered an indicator of functionality and has been shown to be closely correlated with primary infection outcomes [8]. Subjects who subsequently developed persistent viraemia after primary infection had a low level of CD127, when compared with intermediate level expression in those who cleared viraemia [8]. Similarly, a chimpanzee study suggested early expression of CD127 on HCV‐specific T cells was associated with spontaneous clearance of primary infection [9]. In this study, the CD8 memory T cell populations generated following clearance of infection co‐expressed CD127^hi^ and high levels of the anti‐apoptotic marker, Bcl‐2 [9]. CD127 expression is also reported to correlate with upregulation of the chemokine receptors, CCR7 and CXCR4, as well as enhanced IL‐2 production [8]. These phenotypic and functional signatures of HCV‐specific CD8 T cells imply a sub‐population with enhanced polyfunctionality marked by elevated proliferation and cytokine production abilities, which likely make these cells distinct from other central memory and effector memory T cells [10]. Such polyfunctional antigen‐specific CD8 T cells are likely to be associated with the T_SCM_ phenotype [11]. T_SCM_ cells are rare, antigen‐experienced T cells which exhibit enhanced longevity and self‐renewal, as well as multi‐potency, allowing them to reconstitute the entire spectrum of memory and effector T cell subsets [12, 13, 14]. T_SCM_ can be identified using flow cytometry within the naïve (T_N_) population (CD45RA+CCR7+), along with overexpression of CD95. T_SCM_ are now being considered for therapeutic exploitation in adoptive cell transfer therapies and have been associated with long‐term protection against infection in a number of settings, but particularly after Yellow Fever (YF) vaccination [12, 15, 16, 17]. In the context of YF virus vaccination, YFV‐specific CD8+ T_SCM_ cells have been shown to persist for more than 25 years in vaccinated individuals, retaining their ability to self‐renew ex vivo [12]. A study using deuterium labelling of YFV‐specific CD8+ T cells in vivo revealed that these cells mount a rapid and preferential response upon re‐exposure to the pathogen, even a decade post‐vaccination [18]. Additionally, recent research has pointed to a role for CD122+ CD4+ T_SCM_ cells in inhibiting HIV, following immunisation [17]. In contrast, Nagai et al. [15] demonstrated that CD4+ T_SCM_ cells serve as a reservoir for HIV‐1, contributing to the virus's long‐term persistence during antiretroviral therapy. In cases of adult T‐cell leukaemia linked to human T‐cell leukaemia virus type 1 (HTLV‐1), it has been observed that the progenitors of malignant clones are HTLV‐1‐infected CD4+ T_SCM_ cells [16]. Moreover, expanding evidence indicates that autoreactive T_SCM_ cells play a role in autoimmune diseases like rheumatoid arthritis and Type 1 diabetes [19, 20].

There is very limited information about the role of HCV‐T_SCM_ in HCV infection outcomes. Thus, a detailed investigation of the frequencies and functionality of HCV‐T_SCM_ in high‐risk individuals who spontaneously cleared a primary infection episode, and also cleared at least one further infection episode (termed here ‘super‐clearers’), or who subsequently developed chronic infection (termed here ‘clearer‐chronics’), was undertaken.

The hypothesis underlying this project was that circulating frequencies of HCV‐specific T_SCM_ and the functional capacity of those cells via enhanced proliferative, multi‐potency, and self‐renewal abilities are associated with the super‐clearers outcome when compared with the clearer‐chronics re‐infection outcome.

## 2 Results

### 2.1 Clinical Characteristics of Super‐Clearers and Clearer‐Chronics

**FIGURE 1:**
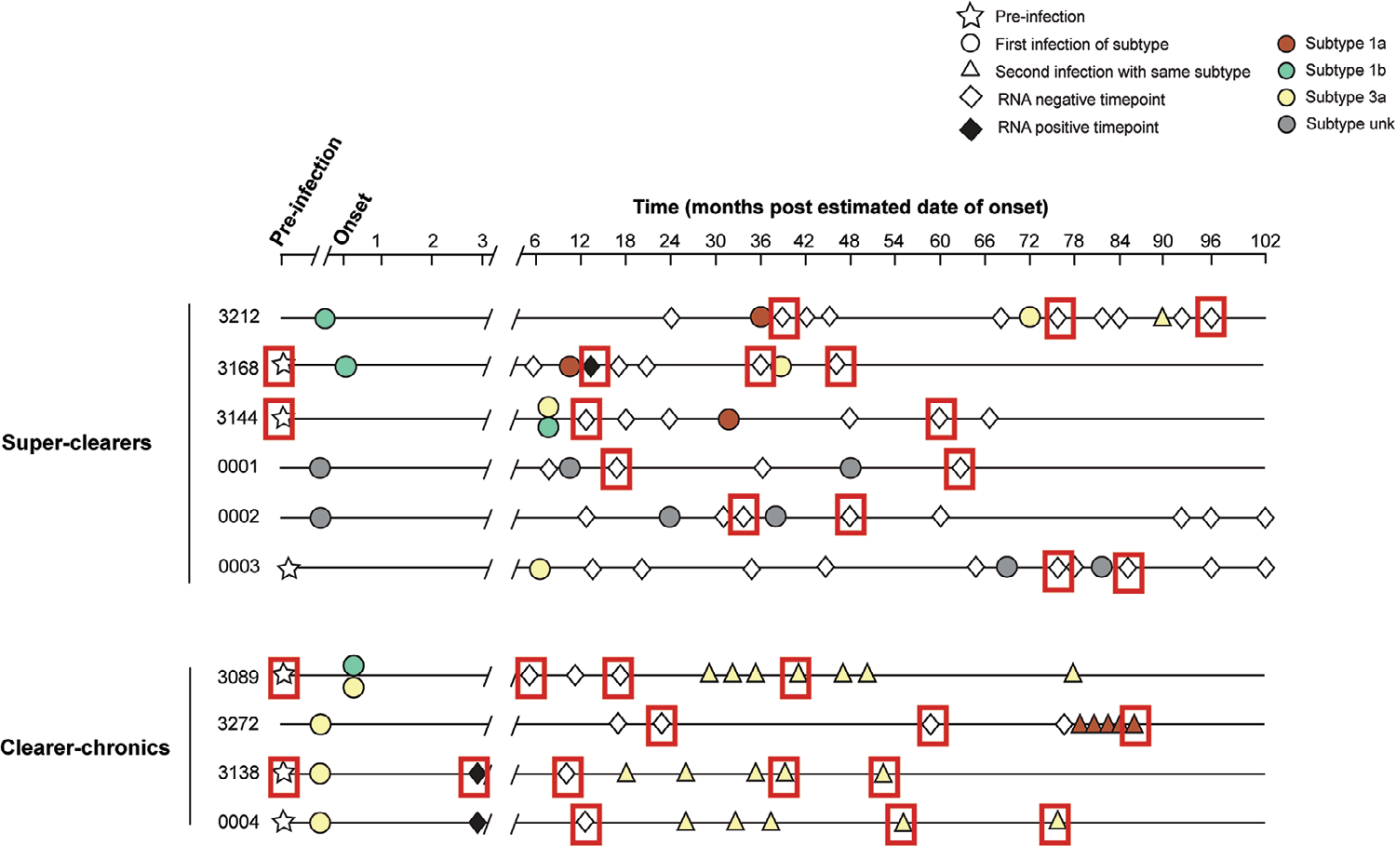
Natural infection history of the super‐clearers and clearer‐chronic subjects included in this study. Data are shown for the *n* = 10 selected subjects identified as having multiple infection episodes: 16 samples from 6 super‐clearers donors and 15 samples from 4 clearer‐chronics donors, including pre‐infection timepoints. The line represents a longitudinal time scale from prior to primary infection, followed by clearance and then to re‐infection and ongoing follow‐up to the last sampling point in months. The colour of the symbol designates the virus genotype/subtype. First and second infections with the same subtype are represented with circles and triangles, respectively. Time points representing RNA‐negative and RNA‐positive results are represented by an empty diamond with a black outline or a black diamond, respectively. Time points where two viral strains were present have two symbols indicated (see key). The timepoints selected for analysis in this study were labelled with red boxes.

A total of 10 subjects satisfying the predefined selection criteria were selected from the HITS‐p and SuperMix cohorts (Tables 1 and 2). Of the 10 subjects, 6 had spontaneously resolved a primary infection episode and also spontaneously cleared at least one further infection episode (i.e., super‐clearers), and 4 had spontaneously resolved a primary infection episode but developed chronic infection with the re‐infection episode (i.e., clearer‐chronics). At least two timepoints (including one at the pre‐re‐infection and one post‐re‐infection timepoint) were selected from the longitudinally collected and stored samples of each subject (Figure 1). The timepoint selected for dextramer screening for each subject for inclusion was based on the anticipated timeframe for maximal frequencies of HCV‐specific memory CD8 T cells ‐ generally around 180 days post‐infection (DPI) to 540 DPI [6, 21]. The natural history of the infection episodes and the genotypes of the viruses infecting the subjects are shown in Figure 1.

**TABLE 1:** Clinical and laboratory characteristics of the HITS‐p cohort (*n* = 6) subjects utilised for HCV‐specific T_SCM_ cell studies.

Outcome of infection	Subject	Age at infection	Gender	HLA type			Epitope & Commercial dextramer	Protein (aa position)	Timepoint after primary infection (days)	Timepoint after re‐infection (days)	HCV RNA	Viral load (IU/mL)	Infecting virus genotype
				A	B	C							
Super‐clearers	3212	21	M	0101	0801	0501	HLA‐A*0201 CINGVCWTV	NS3 (1073–1081)	871	170	neg		1a
				0201	4402	0701			2270	382	neg		3aα
									3128	428	neg		3aβ
	3168	21	M	0201	1501	0303	HLA‐A*0201 CINGVCWTV	NS3 (1073–1081)	−557		neg		−
				2402	4001	0304			382	121	pos	<15	1b
									1074	813	neg		1a
									1444	226	neg		3a
	3144	21	M	0101	1302	0102	HLA‐A*0101 ATDALMTGY	NS3 (1436–1444))	−587				
				3401	5601	0602			611		neg		3a/1b
									1821	475	neg		1a
Clearer‐chronics	3089	27	M	0702	0501	0401	HLA‐B*0702 GPRLGVRAT	E2 (610–618)	−182		neg		
				4402	0702	1501			358		neg		1b/3aα
									755	201	neg		3aβ
									1370	415	pos	534,514	3aβ
	3272	31	F	0101	3501	4010	HLA‐A*0101 ATDALMTGY	NS3 (1436–1444)	803				3a
				2601	0501	6020			1917				3a
									2957	439	pos	279,572	1a
	3138	27	M	0101	0801	0701	HLA‐A*0101 ATDALMTGY	NS3 (1436–1444)	−996				
				3401	4002	0702			902	248	pos	<15	3aα
									1178	524	neg		
									1540	252	pos	345,611	3aβ
									1805	516.5	pos	203,992	3aβ

*Note*: Missing values indicated samples not tested. Information about each subject's HLA restriction, infecting virus genome, viral load was known prior to sample selection and study commencement.

Abbreviations: aa: amino acid; IU: international units

**TABLE 2:** Clinical and laboratory characteristics of the SuperMix cohort (*n* = 4) subjects utilised for HCV‐specific T_SCM_ cell studies.

Outcome of infection	Subject	Age at infection	Gender	HLA type			Epitopes & ULoad dextramers (refer to Tables S2 & S3)	Timepoint after primary infection (days)	Timepoint after each re‐infection (days)	HCV RNA	Viral load (IU/mL)	Infecting virus genotype
				A	B	C						
Super‐clearers	0001	unk	Male	0201	1801	0501	−	unk	287	neg	−	unk
				3002	4402	0501	−	unk	641	neg	−	unk
	0002	unk	Male	−	3501	0401	−	unk	401	neg	−	unk
				−	4402	0501	−	unk	391	neg	−	unk
	0003	19	Male	0201	0702	0102	−	2080	542	neg	−	1a
				0301	2705	0702	−	2374	134	neg	−	1a
Clearer‐chronics	0004	unk	Male	0201	3901	0202	−		−	neg	−	3a
				3201	4402	0702	−	−	232	pos	278760	3a
								−	574	pos	386,174	3a

*Note*: Information about each subject's HLA restriction, infecting virus genome, viral load was known prior to sample selection and study commencement.

Abbreviations: Unk: unknown; IU: international unit.

### 2.2 Greater Proliferative Recall Responses of HCV‐Specific CD8 T Cells Are Associated with Spontaneous Clearance of Re‐Infection in Super‐Clearer

**FIGURE 2:**
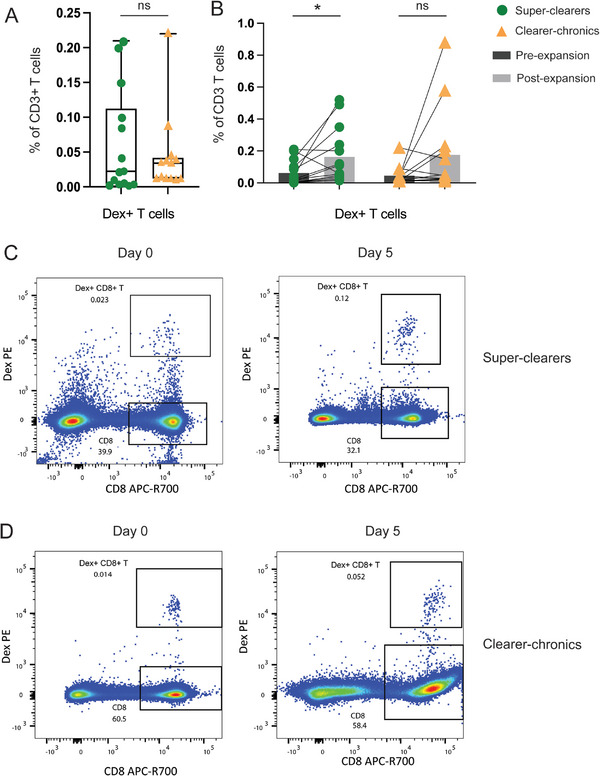
Greater proliferative capacity of Dex+ CD8+ T cells in response to cognate recall in super‐clearers compared with clearer‐chronics after primary infection. (A) Boxplots showing frequencies of total Dex+ CD8+ T cells pooled from all timepoints after primary infection in longitudinally collected samples from super‐clearers and clearer‐chronics. Data are displayed as median (line) with 25th–75th percentiles (box) and compared using the Mann–Whitney *U* test. Data derived from super‐clearers (*n* = 14 samples, 6 donors) and clearer‐chronics (*n* = 13 samples, 4 donors). (B) Connected dot plot of Dex+ CD8+ T cells frequencies before and after stimulation with cognate peptide and IL‐2/IL‐15 in super‐clearers and clearer‐chronics. Each dot represents one timepoint with paired measurements connected by lines. Medians are shown in grey bars. The data are pooled from independent experiments in individual timepoints from super‐clearers (*n* = 14 samples, 6 donors) and clearer‐chronics (*n* = 13 samples, 4 donors) and compared using a Mann–Whitney *U* test. (C, D) Representative flow plot of the frequencies of Dex+ CD8+ T cells in (C) super‐clearer subject 3168 and (D) clearer‐chronic subject 3089 before cognate peptide and IL‐2/IL‐15 stimulation (Day 0), and after stimulation (Day 5).

The frequencies of antigen‐specific CD8 T cells identified by HCV dextramers (Dex+) were recorded from all longitudinally collected timepoints after primary infection (excluding the pre‐infection timepoints) from the selected subjects. Both super‐clearers (*n* = 14 samples, 6 donors) and clearer‐chronics (*n* = 13 samples, 4 donors) had detectable frequencies of HCV‐specific CD8 T cells across multiple timepoints post‐infection. A further analysis combining samples from all timepoints post‐infection for each subject demonstrated that there was no significant difference in the frequencies of total Dex+ CD8+ T cells in super‐clearers compared with clearer‐chronics (Figure 2A).

The proliferative recall capability of total Dex+ CD8+ T cells to recognise cognate peptide and undergo expansion was assessed in both super‐clearers (*n* = 14 samples, 6 donors) and clearer‐chronics (*n* = 13 samples, 4 donors) (Figure 2B, Figure 2C–D). The frequencies of Dex+ CD8+ T cells in super‐clearers demonstrated a significant increase with the cognate peptide and IL‐2/IL‐15 stimulation in vitro, with a median fold increase at 4.55 ± 8.67 (median ± IQR), whereas no significant change was observed in clearer‐chronics (1.87 ± 5.53) (Figure 2B; Figure S3). These data indicate that although the total frequencies of Dex+ CD8+ T cells were comparable between outcome groups, those cells from super‐clearers exhibited a higher proliferative capacity upon cognate recall responses, which might be associated with the varied re‐infection outcome.

### 2.3 Clearer‐Chronic Individuals Exhibit Impaired Memory T Cell Responses Upon Re‐Infection in Comparison to Super‐Clearers

**FIGURE 3:**
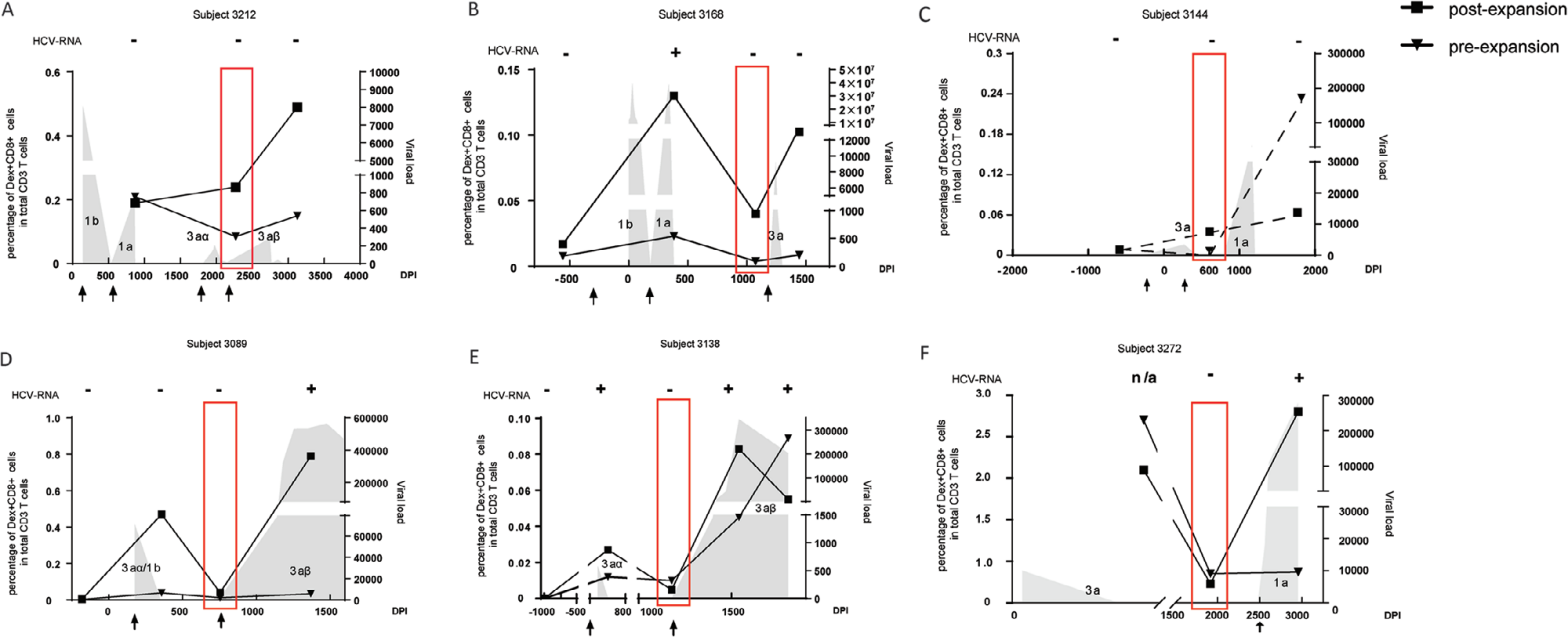
Longitudinal analysis of the frequencies of Dex+ CD8+ T cells at the pre‐expansion and post‐expansion stage in super‐clearers (A–C) and clearer‐chronics (D–F). The kinetics of Dex+ CD8+ T cell recall responses stimulated with cognate peptide and IL‐2/IL‐15 as detected by dextramer staining. Inverted triangles represent the Dex+ CD8+ frequencies at the pre‐expansion stage; the square represents the Dex+ CD8+ frequencies at the post‐expansion stage. The data are derived from independent experiments at individual timepoints from super‐clearers (*n* = 10 samples, 3 donors) and clearer‐chronics (*n* = 12 samples, 3 donors). Grey shaded areas denote the HCV viral load. Each arrow below the *x*‐axis indicates the onset of an HCV infection, and DPI represents the days post‐primary infection. The red boxes indicate the key timepoints before re‐infection. Dashed lines in (C) denote responses where the peptide ATDALMTGY may not have been the appropriate recall peptide for this individual.

Although pooled data from all timepoints revealed that total antigen‐specific CD8+ T cells from super‐clearers and clearer‐chronics responded similarly to cognate peptide stimulation, longitudinal analysis was conducted to further explore potential differences. The frequencies of total Dex+ CD8+ T cells before and after in vitro expansion were evaluated across multiple timepoints in both groups (Figure 3; Figure S5). In super‐clearer subject 3212, who experienced four distinct infection episodes with different viral strains, antigen‐specific CD8+ T cells were detectable both before and after expansion at all relevant timepoints, including just before re‐infection (highlighted in red box, Figure 3A). Similarly, subject 3168, with three distinct infections of varying genotypes, exhibited strong proliferation of Dex+ CD8+ T cells following in vitro stimulation at each post‐infection timepoint, including the third (pre‐re‐infection) timepoint (Figure 3B). In subject 3144 (Figure 3C), Dex+ CD8+ T cells were not detectable at the pre‐infection timepoint but showed increased frequency after the second infection. However, those prevalent cells failed to expand upon stimulation with the cognate peptide (ATDALMTGY) and IL‐2/IL‐15, dropping to ∼0.06% of CD3+ T cells post‐expansion. Sequencing confirmed a mutation in the viral epitope that likely reduced MHC‐peptide binding affinity (Tables S6 and S7), suggesting ATDALMTGY may not have been the appropriate recall peptide for this individual. In subjects 0001, 0002, and 0003 (Figure S5A–C), each with at least two infection episodes, Dex+ CD8+ T cells were detectable at both pre‐ and post‐re‐infection timepoints (highlighted in red box). These cells underwent robust expansion (three‐ to fivefold) following in vitro stimulation, even in the absence of detectable viremia, highlighting their preserved recall capacity.

For clearer‐chronic subject 3089 (Figure 3D), Dex+ CD8+ T cells were present at low frequencies across all four timepoints at the pre‐expansion stage. Notably, at the third timepoint ‐ immediately before re‐infection ‐ Dex+ CD8+ T cells failed to respond to cognate peptide stimulation and remained at a very low frequency, in contrast to the robust expansion observed following the primary (timepoint 2) and secondary (timepoint 4) infections, indicating impaired recall responsiveness during this pre‐re‐infection phase. A similar pattern was observed in subjects 3138 (Figure 3E) and 3272 (Figure 3F), where Dex+ CD8+ T cells were detectable at all post‐infection timepoints before expansion. These cells responded well to in vitro stimulation following the initial infection and during chronic infection. In contrast, at the timepoints immediately preceding re‐infection (highlighted in red box), Dex+ CD8+ T cells were present but failed to expand, revealing a reduced recall potential at a key phase when protection would be most critical. In subject 0004 (Figure S5D), total Dex+ CD8+ T cells were detected at low frequencies across all timepoints before expansion. Although these cells exhibited significant expansion following stimulation at earlier timepoints, they declined sharply to near‐undetectable levels at the chronic phase.

Overall, Dex+ CD8+ T cells were detectable at all examined timepoints following infection in both super‐clearers and clearer‐chronics. In super‐clearers, these cells consistently showed strong proliferative responses to cognate peptide stimulation, including at timepoints preceding re‐infection. In comparison, Dex+ CD8+ T cells from clearer‐chronics also demonstrated expansion after both primary and secondary infections, but their proliferative responses appeared more limited at pre‐re‐infection timepoints (highlighted in red boxes). These observations may reflect subtle differences in the recall potential of HCV‐specific CD8+ T cells between the two groups and raise the possibility that reduced expansion capacity before re‐infection could contribute to differential outcomes.

### 2.4 Super‐Clearers Have Significantly Higher Frequencies of HCV‐T_SCM_ Than Clearer‐Chronics Post‐Expansion

**FIGURE 4:**
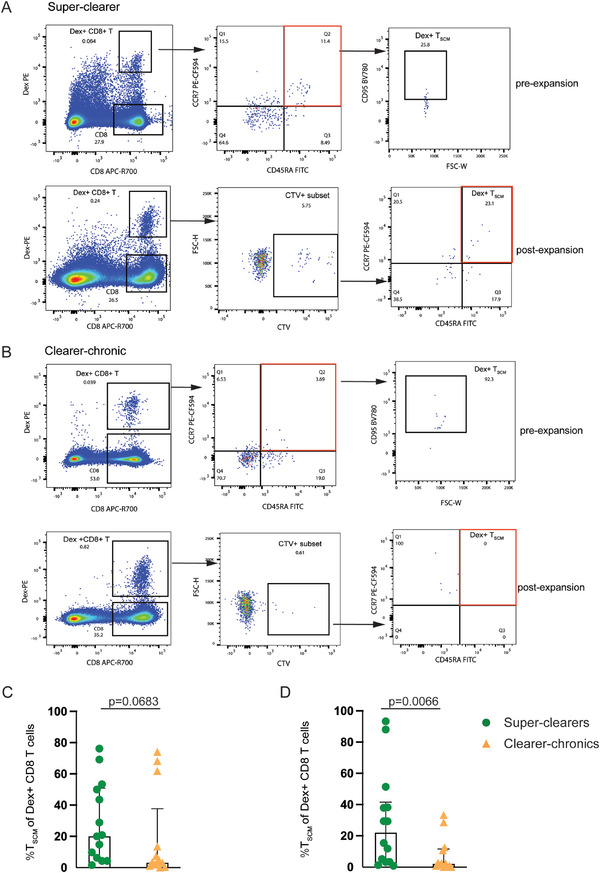
Frequencies of Dex+ T_SCM_ at pre‐/post‐expansion in both super‐clearer and clearer‐chronic subjects. Representative gating strategy for Dex+ T_SCM_ before in vitro expansion (top panel) and post‐expansion (bottom panel) in super‐clearer subject 3212_69M (A) and clearer‐chronic subject 3089_ wk24 (B). For pre‐expansion (day 0), the Dex+ T_SCM_ cells were gated as Dex+CD8+CD45RA+CCR7+CD95+ from thawed PBMCs. For bulk T_SCM_ (CD8+CD45RA+CCR7+CD95) sorting at day 0, 61,938 ± 69,951 (median ± IQR) cells were obtained from super‐clearers (*n* = 14 samples, 6 donors) and 32,076 ± 33,862 from clearer‐chronics (*n* = 13 samples, 4 donors). Sorting efficiency was >95%, starting from ∼5–10 × 10⁶ PBMCs per sample. Special attention was needed for the gating of Dex+ T_SCM_ after expansion (day 5), as only the CTV‐labelled Dex+ T_SCM_ population represents the true HCV‐specific T_SCM_ that had responded and proliferated with the cognate peptide stimulation. Whereas the CTV‐negative population (representing the cells contained in the PBMCs utilised as APCs) was likely to contain antigen‐specific T_CM_, T_EM_, T_EFF_, or even T_N_‐derived T_SCM_ responding to the in vitro stimulation. This strategy excluded CTV‐cells arising from autologous feeder PBMCs, but may potentially underestimate the most proliferative progeny that had diluted CTV below detection. Dot plots summarising the percentage of T_SCM_ among total Dex+ CD8+ T cells at the pre‐expansion stage (C) and post‐expansion stage (D). The data were compared using the Mann–Whitney *U* unpaired test (C, D). Data represented as median with interquartile range are pooled from independent experiments.

Given the observation that total Dex+ CD8+ T cells from clearer‐chronics appeared less responsive at the timepoints preceding re‐infection compared with super‐clearers, we next examined the Dex+ T_SCM_ subset (Figure 4; Table S5).

Representative gating strategies for identifying Dex+ T_SCM_ before and after in vitro expansion are shown for a super‐clearer (subject 3212 at timepoint 69M, Figure 4A) and a clearer‐chronic individual (subject 3089 at week 24, Figure 4B). In pre‐expansion samples, Dex+ T_SCM_ were defined as Dex+CD8+CD45RA+CCR7+CD95+ cells from thawed PBMCs. For post‐expansion analysis, additional care was taken due to the co‐culture of sorted, CTV‐labelled Dex+ T_SCM_ with autologous PBMCs (serving as antigen‐presenting cells). These PBMCs may contain antigen‐experienced T cells (e.g., T_CM_, T_EM_, T_EFF_, or T_N_‐derived T_SCM_) capable of responding to stimulation. However, as these PBMC‐derived cells were not CTV‐labelled, they could be excluded from analysis. Accordingly, only CTV+Dex+CD8+ T cells were considered to represent proliferated T_SCM_ originating from the sorted population (Figure S4).

In the super‐clearer subject (Figure 4A), 23.1% of expanded Dex+ CTV+ CD8+ T cells displayed a T_SCM_ phenotype, whereas no such CTV+ Dex+ T_SCM_ were observed in the clearer‐chronic subject (Figure 4B). Group‐level comparisons across all timepoints are summarised in Figure 4C,D; Table S5. At the pre‐expansion stage, there was no statistically significant difference in the percentage of Dex+ T_SCM_ between super‐clearers and clearer‐chronics (*p* = 0.0683). However, following expansion with cognate peptide and IL‐2/IL‐15, a significantly higher proportion of Dex+ T_SCM_ was observed in super‐clearers (*p* = 0.0066). These findings suggest that HCV‐specific T_SCM_ from super‐clearers are more capable of proliferating and maintaining their phenotype in response to antigenic stimulation compared with those from clearer‐chronics.

### 2.5 Super‐Clearer Display Sustained Self‐Renewal Capacity of HCV‐T_SCM_ Compared with Clearer‐Chronics at Re‐Infection

**FIGURE 5:**
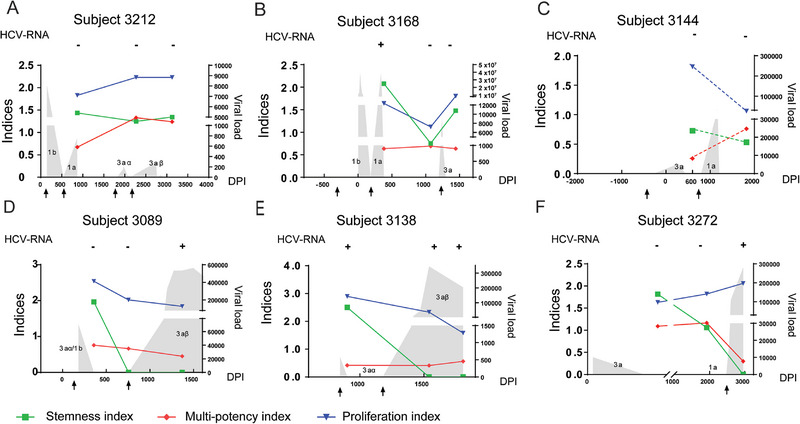
Longitudinal analysis of the proliferation index, multi‐potency index, and stemness index of Dex+ T_SCM_ after expansion in super‐clearers (A–C) and clearer‐chronics (D–F). The inverted blue triangle represents the proliferation index of Dex+ T_SCM_; the red diamond represents the multi‐potency index, while the green square represents the stemness index. The grey shaded areas denote the viral load associated with the HCV infection episodes. Each arrow on the *x*‐axis indicates the onset of the HCV infection episode, and DPI indicates the days post‐infection. The data are derived from independent experiments at individual timepoints from super‐clearers (*n* = 8 samples, 3 donors) and clearer‐chronics (*n* = 9 samples, 3 donors).

To further characterise the functional properties of HCV‐specific T_SCM_, three indices (proliferation, multi‐potency, and stemness [11]) were calculated based on the Dex+ CTV+ T_SCM_ populations in both super‐clearers and clearer‐chronics (Figure 5; Figure S6, and Supporting Information File S2).

**FIGURE 6:**
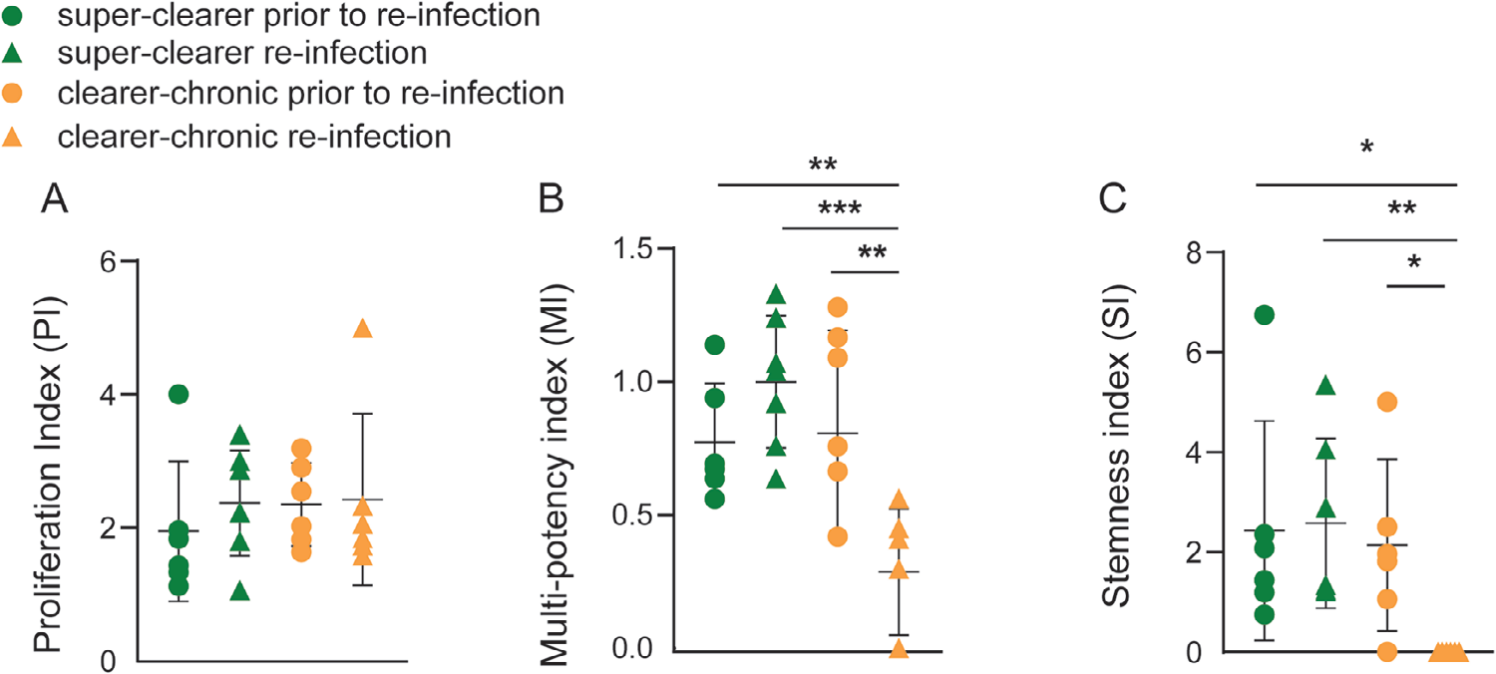
Summary of the proliferation index, multi‐potency index, and stemness index of HCV‐T_SCM_ by infection episodes in super‐clearers and clearer‐chronics. The green colour represents super‐clearers; the orange colour represents clearer‐chronics. The circle represents timepoints before re‐infection; the triangle represents timepoints at or after re‐infection. (A) Proliferation index (PI), (B) multi‐potency index (MI), and (C) stemness index comparisons across timepoints between super‐clearers and clearer‐chronics. The data are derived from independent experiments at individual timepoints from super‐clearers (*n* = 13 samples, 6 donors) and clearer‐chronics (*n* = 12 samples, 4 donors) and compared using unpaired *T*‐tests, bars indicate mean with SD. *p < 0.05; **p < 0.01.

In general, as described above, the HCV‐specific T_SCM_ population in both super‐clearers and clearer‐chronics exhibited good proliferative responses when stimulated with cognate peptide and IL‐2/IL‐15, and their multi‐potency, represented by the multi‐potency index (MI), was also maintained at a relatively stable level, fluctuating moderately along with the viral load (Figure 5). Notably, however, the stemness index (SI) of Dex+ T_SCM_ in clearer‐chronic subjects dropped to zero before re‐infection, some even remained at zero after re‐infection ‐ as observed in subject 3089 (Figure 5D), subject 3138 (Figure 5E), and subject 0004 (Figure S6D). These data are summarised by infection phase in Figure 6, where all timepoints were categorised as ‘prior to re‐infection phase’, or in the ‘re‐infection phase’. While no significant difference in the proliferation index (PI) was observed between the two groups (Figure 6A), a significant reduction in MI was detected in clearer‐chronics during the re‐infection phase compared with super‐clearers, indicating impaired multi‐potency (Figure 6B). Strikingly, HCV‐specific T_SCM_ from clearer‐chronics in the re‐infection phase completely lost their stemness capacity during re‐infection, both in comparison to their own pre‐re‐infection phase and to super‐clearers at all timepoints (Figure 6C).

The stemness property of a cell is characterised by its capacity for self‐renewal and its ability to give rise to differentiated progeny [11]. In this study, a reduction in stemness among HCV‐specific T_SCM_ cells in clearer‐chronic individuals was observed, potentially indicating a diminished ability to maintain the T_SCM_ pool over time. These cells may be more prone to differentiation into other memory subsets, such as T_CM_ and T_EM_, rather than sustaining a self‐renewing population. This trend may help explain, at least in part, the limited immune control of re‐infection observed in this group. While preliminary, these findings raise the possibility that preserved stemness in HCV‐specific T_SCM_ cells could play a role in supporting protective immunity against re‐infection.

## 3 Discussion

This study phenotypically and functionally characterised HCV‐specific T_SCM_ cells using longitudinal samples from individuals who repeatedly cleared HCV infection (“super‐clearers”) and those who progressed to chronic infection after re‐exposure (“clearer‐chronics”). Through a novel in vitro antigen‐driven expansion assay combined with CTV‐labelling, key functional attributes of antigen‐specific T_SCM_ cells were quantified. The findings suggest that the preservation of stemness properties ‐ specifically, self‐renewal and the capacity to regenerate the T_SCM_ pool ‐ may be associated with durable immune protection against HCV re‐infection. Although preliminary, these observations highlight a potential link between impaired T_SCM_ maintenance and susceptibility to re‐infection. The experimental platform established here may also be applicable for investigating antigen‐specific T_SCM_ responses in other chronic viral infections, including HIV and SARS‐CoV‐2.

The antigen‐specific proliferation assays described in this study utilised peripheral blood mononuclear cells (PBMCs) as antigen‐presenting cells (APCs) to present cognate peptides to sorted T_SCM_ cells. While previous studies have typically employed genetically modified dendritic cells transfected with DNA constructs or in vitro‐transcribed RNA [22, 23, 24, 25, 26], artificial APCs [27], or CD40L‐activated B cells [28], such approaches were not feasible here due to limited cell availability per subject and timepoint, as well as restricted access to gamma irradiation for autologous PBMCs [12, 29, 30]. To address this, a gating strategy was developed to isolate CD95+ cells from whole PBMCs. Although residual antigen‐specific T cells within the PBMC population may have proliferated in response to stimulation, and naïve T cells could have undergone peptide‐induced differentiation, these populations were distinguishable in post‐expansion analysis by their lack of CTV labelling. The concentration of CTV was carefully optimised to ensure clear peak resolution and adequate fluorescence intensity across multiple cell divisions during the 5‐day in vitro stimulation. While this conservative approach ensured specificity for progeny derived from the input T_SCM_ fraction, it likely systematically excluded the most proliferative cells that had diluted CTV below the detection threshold. As a result, our analysis likely underestimated the overall self‐renewal and expansion capacity of HCV‐specific T_SCM_. Despite this bias, consistent differences between super‐clearers and clearer‐chronics were observed across multiple donors and infection episodes. Future studies employing complementary approaches such as Ki‐67 expression or lineage‐tracing technologies will be important to capture the full proliferative spectrum of antigen‐specific T_SCM_.

Using this protocol, total HCV‐specific CD8+ T cells exhibited significant expansion in response to cognate antigen stimulation in combination with IL‐2 and IL‐15, with greater proliferative capacity observed in super‐clearers than clearer‐chronics. Interestingly, a previous study reported a higher proliferative capacity of CD8+ T cells in individuals who spontaneously cleared both primary and secondary infections compared with those who progressed to chronic infection following re‐infection [5]. However, those findings were based on stimulation with overlapping HCV peptide pools. Notably, the authors also acknowledged that when cognate peptides were used for stimulation, the proliferative capacity of HCV tetramer+ memory CD8+ T cells was similar between the two groups [5].

Higher frequencies of HCV‐specific T_SCM_ were observed in super‐clearers compared with clearer‐chronics, with functional assessments indicating greater multi‐potency and stemness. These findings suggest that in super‐clearers, HCV‐specific T_SCM_ may have an enhanced ability to self‐renew and differentiate into other memory T cell subsets, potentially contributing to a more effective immune response against re‐infection. This highlights their possible relevance as a target for future prophylactic vaccine strategies. It is worth noting, however, that a NIH‐sponsored phase 1–2 randomised, double‐blind, placebo‐controlled trial of a prime‐boost HCV vaccine in high‐risk PWID did not achieve protection against chronic infection [31]. While the vaccine induced strong HCV‐specific CD8+ T cell responses ‐ reflected by an average peak IFN‐γ ELISpot response of 482 spot‐forming units per million PBMCs ‐ the memory T cell phenotypes generated, including the presence or absence of T_SCM_, were not characterised. These findings underscore the need for further studies to better define the cellular correlates of protective immunity.

Multiple studies in both human and murine models have shown that antigen‐specific CD8+ T cells derived from chronic viral infections often fail to develop into long‐lived memory cells, even after viral clearance, and lack the capacity for homeostatic proliferation in response to IL‐7 and IL‐15 [32, 33, 34, 35]. The findings from this study offer a potential mechanistic insight into this phenomenon, suggesting that the observed loss of stemness in HCV‐specific T_SCM_ may underlie their diminished ability to self‐renew and to sustain the antigen‐specific memory T cell compartment. In line with this, the study by Hensel et al. [35] identified a memory‐like subset of HCV‐specific CD8+ T cells with high CD127 expression that persisted after antigen removal but retained a residual exhaustion‐associated transcriptional program, described as a molecular ‘scar’. While this study provided valuable molecular insight, functional capacity was not directly assessed. The present study has provided complementary evidence, demonstrating that the stem‐like populations can persist across different clinical contexts, and that the presence or absence of such a scar may be associated with the observation that super‐clearers maintained functional T_SCM_ stemness with repeated viral clearance, whereas clearer‐chronics lost stemness and failed to control re‐infection.

In certain subjects and infection episodes, sequencing of autologous viral epitopes revealed mismatches with the reference sequences used in the commercial MHC‐I dextramers and cognate peptides applied in the antigen‐specific proliferation assays. This discrepancy introduces a potential limitation in the interpretation of the findings. Nonetheless, the MHC‐I dextramers incorporating reference epitopes were still substantially recognised by antigen‐specific T cells at the majority of selected timepoints in both super‐clearers and clearer‐chronics.

This study has several limitations. First, due to limited cell availability, the cytokine‐producing capacity of HCV‐specific T_SCM_ cells could not be assessed using ex vivo assays. Second, the inherently low frequency of HCV‐specific T_SCM_ cells posed technical challenges, even with the established in vitro antigen‐specific proliferation assay. A minimum of 32,000 sorted bulk T_SCM_ cells from 5 to 10 million PBMCs per sampling point was required to ensure sufficient events for reliable flow cytometric analysis. For more detailed characterisation of subpopulations, up to 25 million PBMCs per sample would be needed. To mitigate this limitation, the newly developed ULoad dCODE dextramer technology was adopted, allowing for the pooling of multiple immunodominant epitopes per subject and thus improving the detection sensitivity of HCV‐specific T cells. Lastly, and most critically, the study was constrained by a small number of re‐infection subjects and incomplete sampling across timepoints. Given that most HCV infection episodes are asymptomatic, identifying such cases is challenging outside of large prospective cohort studies. This difficulty has been further compounded by the widespread use of highly effective direct‐acting antivirals (DAAs). While the total cohort comprised only 10 donors, our analysis leveraged multiple timepoints per subject, thereby providing valuable within‐subject longitudinal comparisons across infection episodes. Validation in larger cohorts will be essential to confirm these observations and to establish the generalisability of T_SCM_ as correlates of protective immunity in HCV and other chronic viral infections. Importantly, these limitations provide a rationale for why functional indices were applied as integrative summary measures, capturing multiple attributes in settings where cell numbers were restricted. While these indices cannot substitute for direct functional assays, their convergence with the raw data strengthens confidence in the conclusions and provides a framework for future studies to build upon.

Sustained stemness in HCV‐specific T_SCM_ was associated with repeated viral clearance, suggesting that these cells may contribute to durable protective immunity. Similar patterns have been reported in other viral settings, including the long‐term persistence of YFV‐specific T_SCM_ with recall capacity decades after vaccination [12, 18] and the restoration of CD8+ T_SCM_ frequencies during antiretroviral therapy in HIV infection, where higher levels correlated with improved immune control [15, 36]. These observations provide a rationale for incorporating T_SCM_‐targeted strategies into vaccine design, where cytokine supplementation (IL‐7, IL‐15, IL‐21), cognate peptide priming, or transient blockade of type I interferon signalling [37] could be leveraged to enhance the generation and longevity of protective responses. The importance of T_SCM_ is further underscored by evidence that the frequency of CD8+CD45RA+CCR7+ cells within CAR‐T products predicts their in vivo expansion and persistence [38], and that CD19‐specific CAR‐modified T_SCM_ generated under IL‐7/IL‐21/TWS119 conditions display superior metabolic fitness and long‐lasting antitumor activity [39]. Together, these insights, aligned with the present findings in HCV, point to virus‐specific T_SCM_ as clinically relevant correlates of immune protection and highlight their potential as a target for rational vaccine design and adoptive immunotherapies aimed at achieving durable immune control in both chronic viral infections and cancer.

## 4 Methods

### 4.1 Sex as a Biological Variable

Male and female patients were both included in this study; 9/10 are male and 1/10 is female. Sex was not considered a biological variable in this study.

### 4.2 Subjects and Samples

High‐risk people who inject drugs (PWID) with stored PBMCs and documented primary and re‐infection outcomes were selected from the Hepatitis C Incidence and Transmission Study in prisons (HITS‐p) cohort [40], and from the SuperMix cohort [41, 42, 43]. From both cohorts, risk behaviour data and blood samples were collected approximately every six months to screen for seroconversion and for HCV RNA positivity. The estimated date of primary infection for those detected pre‐seroconversion was calculated by subtracting the pre‐seroconversion‐post‐viraemia window period of 51 days from the midpoint between the preceding HCV‐RNA positive/HCV‐antibody negative time point and the first seropositive time point, or for those detected by seroconversion, as the mid‐point between the last seronegative and first seropositive time point [44]. Infection outcome was determined by considering whether each individual continued to test positive for viral RNA at six months following initial detection of viraemia in the absence of antiviral treatment.

### 4.3 Study Approval

Ethical approval was obtained from: Human Research Ethics Committee of Justice Health (reference number G304/11), New South Wales Department of Corrective Services (11/103694), the University of New South Wales (HREC HC11579 and HREC HC13237), and Victorian Department of Health HREC (project 02/05). Written informed consent was obtained from all participants. Human peripheral blood obtained in buffy coats from healthy donors from the Australian Red Cross Blood Service was utilised as negative controls for analyses included in this study. All donors gave written informed consent, and the laboratory studies were approved by the Human Research Ethics Committee of the University of New South Wales (HC190074).

The selection of subjects from HITS‐p and SuperMix cohorts was performed based on similar selection criteria. As illustrated in Figure S1, subjects were first identified by their infection outcomes: (1) super‐clearers: individuals who cleared primary infection and at least one further infection episode; (2) clearer‐chronics: individuals who spontaneously cleared the primary infection, but developed chronic infection with the re‐infection episode. Subsequently, subjects were selected based on the available stored PBMC vials at designated timepoints. Further refinement in the selection process was achieved by identifying subjects with HLA alleles compatible with the broad panel of HCV‐specific MHC Class I dextramers. The final inclusion criterion was the presence of detectable frequencies of HCV‐specific (Dex+) CD8 T cell responses on dextramer screening, as well as the availability of the corresponding samples.

### 4.4 HLA Typing

Molecular HLA typing was performed at the Institute of Immunology and Infectious Diseases in Perth, Australia, using second‐generation sequencing of HLA‐A/B/C genes.

### 4.5 PBMC Isolation and Cryopreservation

Preparation of PBMCs was performed as previously described with minor modifications [45]. Briefly, PBMCs were isolated from sodium heparin anti‐coagulated buffy coats using Lymphoprep (STEMCELL Technologies, Tullamarine, Australia) by density gradient centrifugation. The cells were washed once in sterile phosphate‐buffered saline (PBS), and then in RPMI‐1640 (Gibco BRL, Waltham, USA), followed by resuspension at 5–12 × 10^6^/mL in complete medium comprised of RPMI‐1640 supplemented with 20% (v/v) heat‐inactivated Fetal Bovine Serum (HI‐FBS; Gibco BRL), 100 U/mL penicillin, 100 µg/mL streptomycin, and 2 mM ʟ‐glutamine. Cells were cryopreserved in 90% complete medium/10% dimethyl‐sulfoxide (DMSO) (v/v). Alternatively, following isolation of PBMCs using Lymphoprep, cells were washed once in sterile Dulbecco's phosphate‐buffered saline (DPBS), and cryopreserved in 90% fetal calf serum (v/v) /10% DMSO (v/v) at 5–12×10^6^ cells per vial.

### 4.6 Detection and Phenotypic Analysis of HCV‐Specific CD8+ T Cells via Commercial MHC I Dextramer Staining or ULoad dCODE Dextramer Staining

Detection of the HCV‐specific CD8+ T cell populations was performed on thawed PBMCs with HLA Class‐I dextramers (Table S1) or ULoad dCODE dextramer (Table S2) (Immudex, Copenhagen, Denmark) in flow cytometric analyses.

For subjects from the HITS‐p cohort, potential immunodominant HCV epitopes for each subject's HLA type were selected using the IEDB database (https://www.iedb.org). The panel of peptide epitopes (up to 1000 per subject) were synthesised with >95% purity (Mimotopes, Mulgrave, Australia) and diluted in DMSO to 1 mM before screening of samples by matrix ELISpot as previously described [46]. Customised dextramers were then synthesised by Immudex for each confirmed ELISpot‐positive peptide before screening of samples with individualised dextramer for HCV‐specific CD8+ T cell frequencies (Tables S1 and S3).

For subjects from the SuperMix cohort (i.e., subjects 0001, 0002, 0003, and 0004), detection of the HCV‐specific CD8+ T cell populations was performed on PBMCs with ULoad dCODE MHC‐I dextramer pools (Immudex) (Tables S1 and S3). We adopted this newly developed dextramer technology, aiming to maximise the detection of the frequencies of HCV‐specific CD8+ populations. Similarly, HCV likely immunodominant epitopes for each HLA type were selected using the IEDB database (https://www.iedb.org). The panel of epitope peptides (up to 12 per subject) with >95% purity was ordered from Mimotopes and diluted in DMSO to 1 mM. Specifically, HLA monomers (easYmer) for A*01:01, A*02:01, A*03:01, A*11:01, A*24:02, B*07:02, B*08:01, B*40:01, and B*44:02 were purchased from Immudex. Preparation of easYmer monomers and synthesis of each ULoad dCODE dextramer was performed according to the manufacturer's protocol. Individual dextramer cocktails were prepared immediately before staining. For each subject, a panel containing up to 13 individual ULoad dCODE dextramers was pooled as a dextramer cocktail. Each cocktail had 2 µL of each HLA‐compatible barcoded dextramer and 0.2 µL 100 µM biotin per dextramer pre‐mixed to block free binding sites.

Thawed PBMCs were washed once with RPMI and PBS, then stained with PE‐labelled, subject HLA‐matched dextramers for 20 min at room temperature in the dark. After incubation, the cells were washed twice with PBS containing 1% BSA. Next, cells were stained for viability with live/dead fixable yellow 405 nm excitation stain (Invitrogen, California, United States) for 30 min at 4°C. After incubation, cells were washed twice with PBS containing of 1% BSA, then stained with the following antibody mix at 4°C for 25 min (all antibodies were purchased from BD Bioscience): CCR7‐PE‐CF594 (clone 150503), CD3‐Brilliant Violet 480 (clone UCHT1), CD19‐PE‐Cy5 (clone HIB19), CD8‐allophycocyanin (APC)‐R700 (clone SK1), CD45RA‐fluorescein isothiocyanate (FITC) (clone HI100), and CD95‐Brilliant Violet 786 (clone DX2). Finally, cells were washed twice and resuspended in 300 µL of PBS/1% of BSA for flow cytometry.

Resuspended PBMCs were analysed on a LSR Fortessa X‐20 flow cytometer with 5 lasers (BD Biosciences). Generally, at least 40,000 events were collected per sample for dextramer‐positive CD8 T cell screening. Anti‐Mouse Ig, κ (BD Biosciences) compensation beads were used as the single‐fluorophore control for every antibody used in the staining panel. Fluorescence‐minus‐one (FMO) controls were utilised to set the gating strategy for markers with non‐discrete expression profiles, such as CD95. The gating strategy used for dextramer‐positive CD8 T cells was first by gating the lymphocyte population with forward scatter (FSC) against side scatter (SSC), then singlet cells were gated with the height (FSC‐H) versus area (FSC‐A), followed by live cells with the exclusion of the viability marker, LIVE/DEAD Fixable/Yellow. HCV‐specific CD8 T cells were subsequently identified as within the CD3‐positive, CD19‐negative, and dextramer‐positive cell population. The phenotypic analysis on HCV‐specific CD8 T cells was performed with the surface staining of CD45RA, CCR7, and CD95.

### 4.7 Bulk Sorting of T_SCM_ and CellTrace Violet Labelling

CD8 T_SCM_ cells were sorted from thawed PBMCs based on surface co‐expression of CCR7, CD45RA, and CD95 using a FACSAria III cell sorter (BD Biosciences, San Jose, USA). The desired cell populations were directly sorted into FACS tubes containing 1 mL of complete medium. For super‐clearers (*n* = 14 samples, 6 donors), a median of 61,938 T_SCM_ (IQR 69,951) was recovered, and for clearer‐chronics (*n* = 13 samples, 4 donors), a median of 32,076 T_SCM_ (IQR 33,862) was recovered. Post‐sorting analysis of purified subsets confirmed >95% purity of the T_SCM_ population, with starting material of ∼5–10 × 10⁶ PBMCs per vial. The bulk T_SCM_ population was centrifuged at 340 rpm for 5 min to pellet the cells. CellTrace Violet (CTV; C34557, ThermoFisher, Waltham, USA) stock solution was prepared by adding the appropriate amount of DMSO to the CellTrace reagent vial. A final working solution of 5 µM was prepared, and 1 µL of this CTV stock solution was used in each mL of cell suspension in PBS. This concentration of CTV was titrated to achieve bright initial staining, ensuring that fluorescence remained detectable even after multiple divisions (Figure S4). The mixture was incubated at 37°C for 20 min, protected from light. After incubation, five times the original staining volume of complete culture medium was added to the cells for 5 min to remove any free dye remaining in the solution. The cells were pelleted by centrifugation at 340*g* for 5 min before the washing step was repeated to completely remove any remaining dye, and final resuspension of the cells was performed in fresh complete medium.

### 4.8 Gating and Sorting Strategy for Residual Autologous Feeder Cells

To sort residual autologous feeder cells to present the cognate peptide to the bulk T_SCM_, a specific gating strategy (Figure S2) was utilised to sort the whole PBMCs without affecting T_SCM_ sorting. Firstly, a CD95+ gate was made for the total PBMC T_SCM_ were sorted in bulk from this CD95+ population by sequential gating as Fix/Yellow‐, CD3+, CD19‐, CD8+, CD45RA+, CCR7+, and CD95+ (Figure S2, upper panel). The residual autologous cells were sorted through a CD95‐ gate (Figure S2, lower panel)—through an inverted gating strategy. This population contained the majority of T_N_ cells, along with some T_CM_, T_EM_, and T_EFF_ cells. The sorted CD95^−^ cells served as autologous feeder cells for the T_SCM_ in the HCV‐specific proliferation assay.

### 4.9 In Vitro Stimulation, Proliferation, and Multi‐Potency Assays on HCV‐Specific T_SCM_

For the in vitro stimulation, bulk‐sorted, CTV‐labelled T_SCM_ cells (∼20,000 cells per well) were mixed thoroughly with 0.5 × 10^6^ autologous PBMCs in complete medium at a final volume of 200 µL. The mixture was then cultured in 96‐well U‐shaped bottom plates with cognate peptide 1 µg/mL (Mimotopes), recombinant human IL‐2 at 50 ng/mL (STEMCELL) and recombinant human IL‐15 at 50 ng/mL (Biolegend, San Diego, USA) for 5 days. As a positive control, 0.25 × 10^6^ autologous PBMCs were stimulated with ImmunoCult Human CD3/CD28 T‐cell activator (25 µL/mL, STEMCELL) for 5 days. Fresh complete culture medium and IL‐2/IL‐15 were half‐replaced after 3 days of culture. Cells were harvested after culture, washed twice with PBS and then stained with HLA‐matched dextramers, Fix/Yellow, and the antibody mix described above for full‐panel staining. Cells were first gated as CTV‐labelled and CTV‐unlabelled populations.

T‐cell assays were designed and reported in accordance with the MIATA (minimal information about T‐cell assays) guidelines.

### 4.10 Calculation of PI, MI, and SI

The proliferation modelling was established on the CTV‐labelled cell populations with the PI of HCV‐specific T_SCM_ calculated based on the established proliferation model (https://docs.flowjo.com/flowjo/experiment‐based‐platforms/proliferation/). Briefly, *G*_0_ represents the number of undivided (generation 0) cells, and *G*_n_ denotes the number of cells in division generation n (e.g., *G*_1_, *G*_2_, *G*_3_). The number of divided cells was defined as the sum of all *G*_n_ for *n*≥1.

The total number of divisions was calculated as follows:

$$\mathrm{Total}\;\mathrm{divisions}=\sum_{n=1}^{N}\left(\frac{G_{n}}{2_{n}}\times n\right)$$

The estimated number of cells that entered division was calculated as:

$$\mathrm{Cells}\;\mathrm{in}\;\mathrm{divisions}=\sum_{n=1}^{N}\frac{G_{n}}{2^{n}}$$

The PI was defined as:

$$\mathrm{PI}\;=\frac{\mathrm{Total}\;\mathrm{divisions}}{\mathrm{Cells}\;\mathrm{in}\;\mathrm{divisions}}$$

Worked example:

In the representative Dex+ CTV labelled population after in vitro expansion (Figure S4D,G), the CTV peaks contained: G1: 0 cell, G2: 3 cells, G3: 4 cells, G4: 0 cell, G5: 0 cell, G6: 6 cells.

Calculations:

$$\mathrm{Cells}\;\mathrm{in}\;\mathrm{division}=\frac{3}{2^{2}}+\frac{4}{2^{3}}+\frac{6}{2^{6}}\approx 1.34$$

$$\mathrm{Total}\;\mathrm{divisions}=\frac{3}{2^{2}}\;\times \;2+\frac{4}{2^{3}}\;\times \;2+\frac{6}{2^{6}}\;\times \;2\;\approx \;3.56$$

$$\mathrm{PI}\;=\frac{3.56}{1.34}\approx 2.66$$

The phenotypic changes in the progeny of CTV‐labelled HCV‐specific T_SCM_ were determined using FlowJo software (Version 10.7.1, Ashland, USA). A MI and SI were derived to provide a quantitative measure of the multi‐potency (i.e., the quantified ability to generate progeny with T_CM_, T_EM_, T_EFF_ and T_N_ phenotypes) and self‐renewal ability (i.e., the ability to regenerate the T_SCM_ phenotype) after stimulation. The calculation of self‐renewal index (SRI) and MI was based on the measures previously defined by Gattinoni et al. [11] with minor modifications: SRI = 2PI×P_SCM_, where PI is the PI and P_SCM_ is the percentage of T_SCM_ generated after stimulation. MI was calculated as the net entropy of the progeny T cell subsets, where *p* is the percentage of a given T cell subset generated after stimulation. SI was calculated by multiplying SRI and MI.

$$\mathrm{MI}=\sum_{i}^{n}-pi\ln pi$$

In the representative Dex+ CTV labelled population after in vitro expansion (Figure S4C), the proportions of progeny subsets were: T_SCM_: 41.2%, T_CM_: 2.9%, T_EM_: 55.9%, T_EFF_: 0%.

Additionally, the PI was 2.66, and T_SCM_ represented 41.2% of the divided progeny (i.e., P_SCM_ = 0.41).

Calculations:

$$\begin{array}{c} \mathrm{MI}=-[(0.41\times \ln0.41)+(0.03\times \ln0.03)+(0.56\times ln0.56)\\ \;\;+\;(0\times \ln0)]\approx 0.79\\ \mathrm{SI}=2\times 2.66\times 0.79=4.2 \end{array}$$

### 4.11 Statistical Analysis and Calculations

Statistical analyses were performed using Prism (GraphPad Software v8.4.1). Data were expressed as the median with minimum to maximum range and analysed with the Mann–Whitney *U* unpaired test. For PI, MI, and SI analysis, an unpaired *t*‐test was used to compare means between groups. For each analysis, *p* values ≤0.05 were considered statistically significant, and significance was denoted as follows: *p < 0.05, **p < 0.01, ***p < 0.001, ****p < 0.0001.

## Author Contributions

Conceptualisation: Yanran Zhao, Heidi Drummer, Rowena A. Bull, Fabio Luciani, Andrew R. Lloyd. Methodology: Yanran Zhao, Andrew R. Lloyd. Investigation: Yanran Zhao, Elizabeth Keoshkerian, Hui Li. Visualisation: Yanran Zhao. Supervision: Rowena A. Bull, Fabio Luciani, Andrew R. Lloyd. Cohort design, collection, and management: Rachel Sacks‐David, Irene Boo, Paul Dietze, Margaret Hellard, Heidi Drummer. Writing – original draft: Yanran Zhao, Andrew R. Lloyd. Writing – review & editing: Yanran Zhao, Heidi Drummer, Andrew R. Lloyd.

## Funding

This work was supported by the Australian National Health and Medical Research Council (NHMRC) Program Grant APP1150078 (to A. R. L.), APP545891, APP1126090, APP2023690, APP1001144; NHMRC Career Development Fellowships APP1084706 (to R. A. B.) and APP1128416 (to F. L.); and NHMRC Practitioner Fellowship APP1137587 (to A. R. L.)

## Conflicts of Interest

The authors declare no conflicts of interest.

## Supporting information




**Supporting File 1**: eji70098‐sup‐0001‐SuppMat.pdf.

**Supporting File 2**: eji70098‐sup‐0002‐SuppMat.xlsx.

**Supporting File 3**: eji70098‐sup‐0003‐SuppMat.xlsx.

## Data Availability

All data supporting the findings of this study are available within the article and its supplementary information files.
