# Supplementary material for: Functional Antigen‐Specific CD8 TSCM Responses Are Associated with Repeated Clearance of Hepatitis C Virus Infection
Source: Eur J Immunol. 2025 Dec 18;55(12):e70098. doi: 10.1002/eji.70098 (PMC12712888; doi:10.1002/eji.70098)
Supplement: Supplementary file 1 — Supporting File 1: eji70098‐sup‐0001‐SuppMat.pdf. [file EJI-55-e70098-s001.pdf]

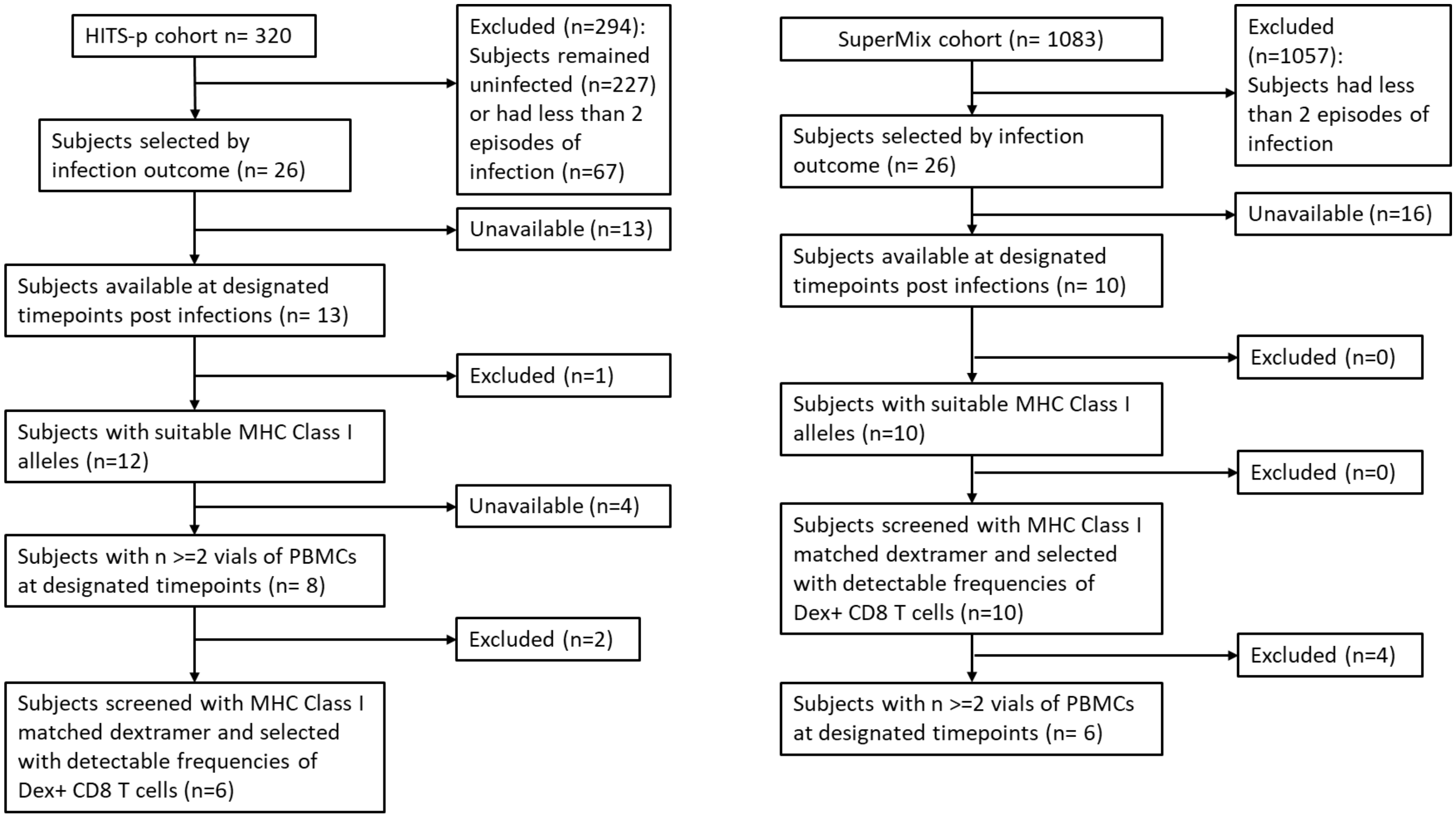

***Supplementary Figure 1 Consort diagram for study inclusion for subjects from the HITS-p cohorts (35) and* SuperMix *cohorts (37)*.**

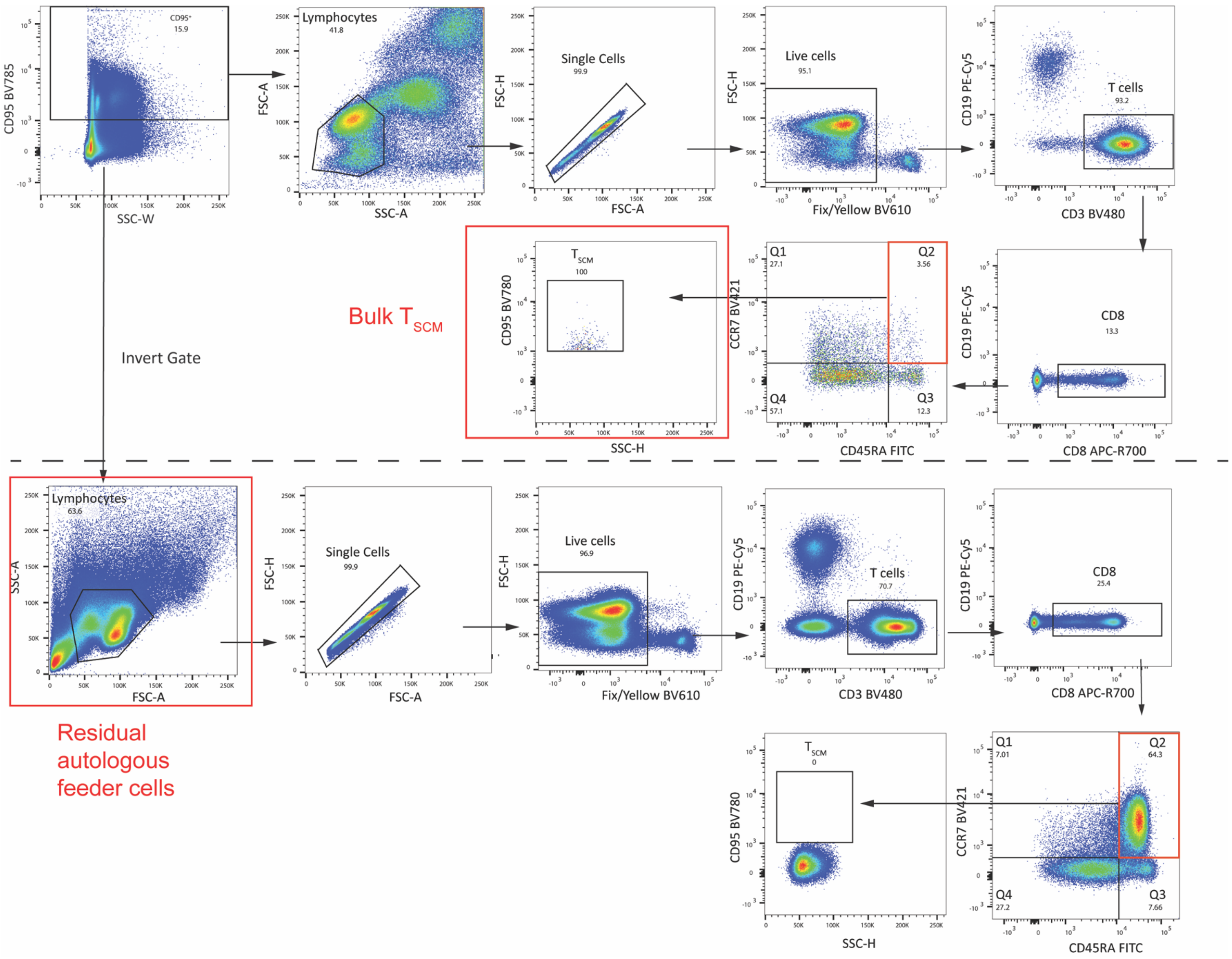

CD95 BV785
SSC-W
CD95+
15.9
Lymphocytes
41.8
FSC-A
SSC-A
Single Cells
99.9
FSC-H
FSC-A
Live cells
95.1
FSC-H
Fix/Yellow BV610
T cells
93.2
CD19 PE-Cy5
CD3 BV480
Invert Gate
Bulk T_{SCM}
T_{SCM}
100
CD95 BV780
SSC-H
Q1
27.1
Q2
3.56
Q3
12.3
Q4
57.1
CCR7 BV421
CD45RA FITC
CD8
13.3
CD19 PE-Cy5
CD8 APC-R700
Lymphocytes
63.6
SSC-A
FSC-A
Residual autologous feeder cells
Single Cells
99.9
FSC-H
FSC-A
Live cells
96.9
FSC-H
Fix/Yellow BV610
T cells
70.7
CD19 PE-Cy5
CD3 BV480
CD8
25.4
CD19 PE-Cy5
CD8 APC-R700
T_{SCM}
0
CD95 BV780
SSC-H
Q1
7.01
Q2
64.3
Q3
7.66
Q4
27.2
CCR7 BV421
CD45RA FITC

Supplementary Figure 2 Gating strategy for sorting bulk T_{SCM} and autologous feeder cells from whole PBMCs

The CD95+ gate was first made on total PBMCs against SSC-W. Bulk T_{SCM} were sorted sequentially from the PBMCs with positive CD95 expression (upper panel). All remaining CD95- cells were gated out (bottom panel) and sorted for use as the autologous feeder cells for cognate peptide stimulation of antigen-specific T_{SCM}. The bulk T_{SCM} percentage in the feeder cell population was examined with the same gating strategy (bottom panel). As a marker with non-discrete expression pattern, the gating strategy for CD95 was determined by FMO control.

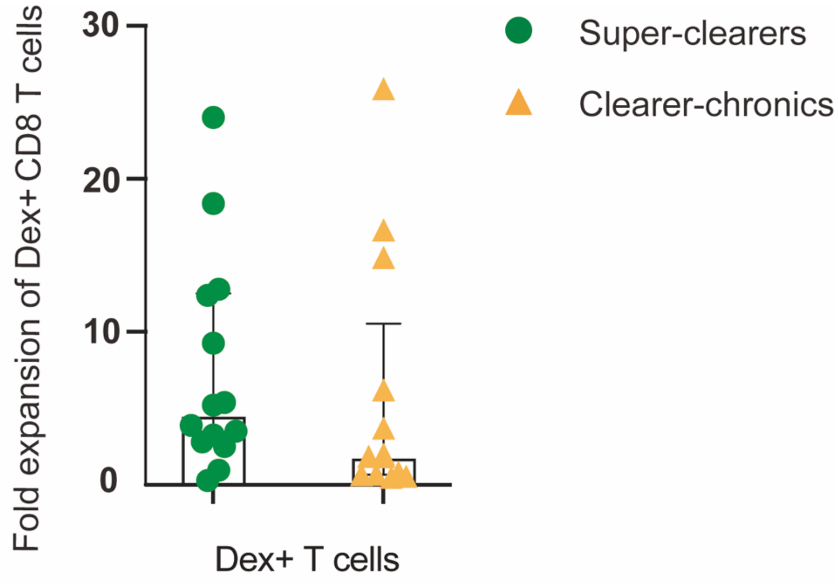

Supplementary Figure 3 Bar graphs of the fold expansion of total Dex+ CD8 T cells after stimulation with cognate peptide and IL-2/IL-15 in super-clearers and clearer-chronics. *The fold expansion was determined by dividing the number of Dex+ CD8 T cells at the post-expansion stage by the number at the pre-expansion stage. The data are derived from independent experiments at individual timepoints from super-clearers (n = 14 samples, 6 donors) and clearer-chronics (n = 13 samples, 4 donors). Data represented as median (line) and 25th to 75th percentile (box) are pooled from independent experiments.*

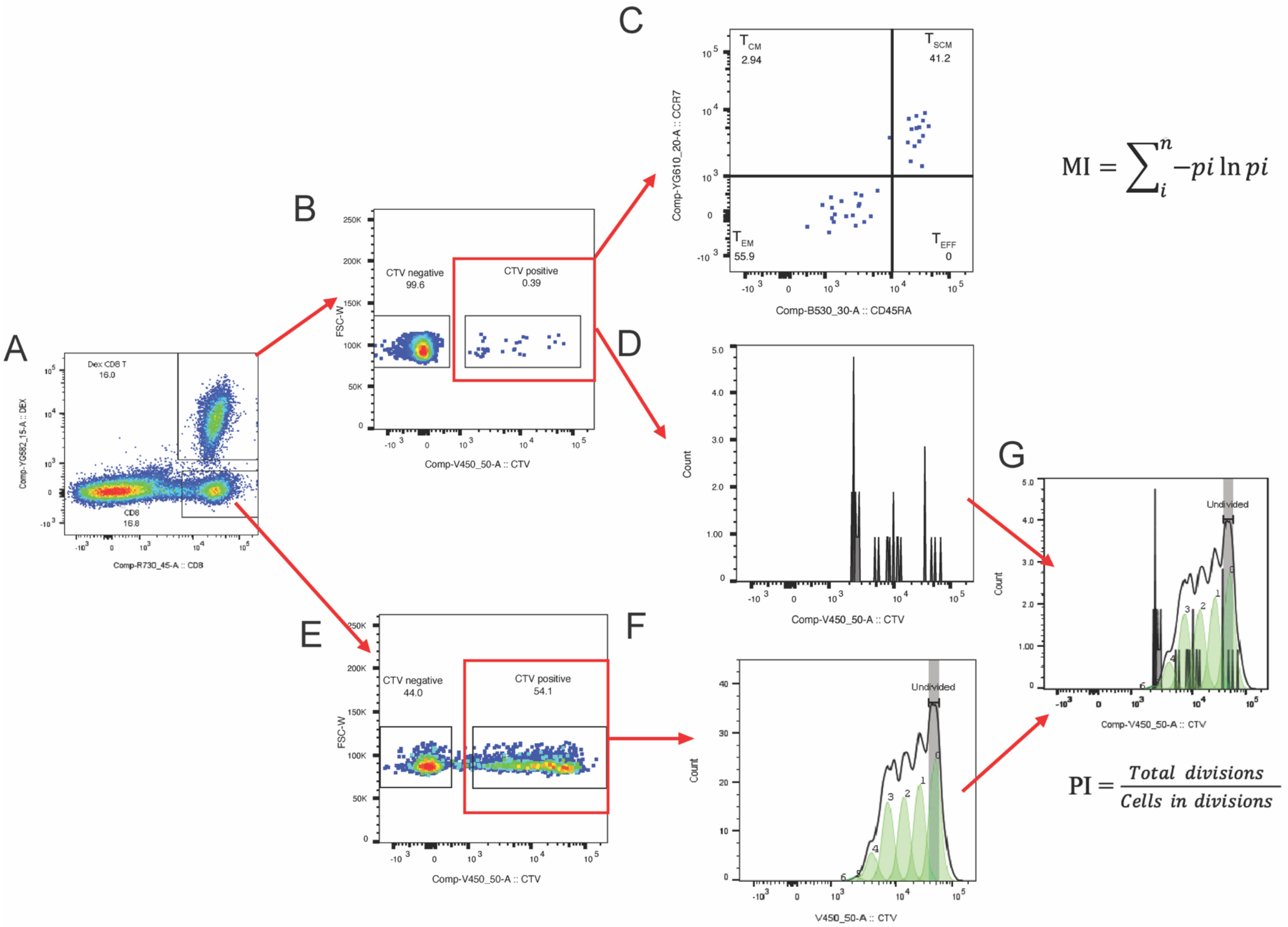
A
Dex CD8 T
16.0
CD8
16.8
Comp-YG582_15-A :: DEX
Comp-R730_45-A :: CD8
B
CTV negative
99.6
CTV positive
0.39
FSC-W
Comp-V450_50-A :: CTV
C
T_{CM}
2.94
T_{SCM}
41.2
T_{EM}
55.9
T_{EFF}
0
Comp-YG610_20-A :: CCR7
Comp-B530_30-A :: CD45RA
D
Count
Comp-V450_50-A :: CTV
E
CTV negative
44.0
CTV positive
54.1
FSC-W
Comp-V450_50-A :: CTV
F
Undivided
Count
V450_50-A :: CTV
G
Undivided
Count
Comp-V450_50-A :: CTV
$$\mathrm{MI} = \sum_{i}^{n} -pi \ln pi$$
$$\mathrm{PI} = \frac{Total\ divisions}{Cells\ in\ divisions}$$

Supplementary Figure 4. Representative flow cytometry plots illustrating the calculation of the proliferation index of CTV-labelled, proliferating Dex+ populations.

(A) Total CD8+ T cells after 5 days of in vitro expansion with cognate peptide and IL-2/IL-15. (B) CTV fluorescence intensity of Dex+ CD8+ T cells showing two subsets: CTV-negative, and CTV-positive. This CTV-positive gating avoids contamination of the feeder-derived responded cells but may potentially underestimate the most proliferative progeny that had diluted CTV below detection. (C) The expression of CD45RA and CCR7 on CTV-positive populations was used to define T_{SCM}*,* T_{CM}*,* T_{EM}*, and* T_{EFF} *subpopulations generated after in vitro expansion. MI was calculated as the net entropy of the progeny T cell subsets where p is the percentage of a given T cell subset generated after stimulation. (D) Histogram of CTV fluorescence counts for total CTV-labelled Dex+ cells. (E) CTV fluorescence intensity of Dex- CD8+ T cells showing two subsets: CTV-negative, and CTV-positive. (F) Histogram of CTV fluorescence counts for total CTV-labelled Dex- cells. (G) Overlay of histograms from panels D and F indicating the number of peaks corresponding to Dex+ CTV-labelled cells. PI was calculated for these Dex+ CTV-labelled cells as the total number of divisions divided by the number of cells that entered division.*

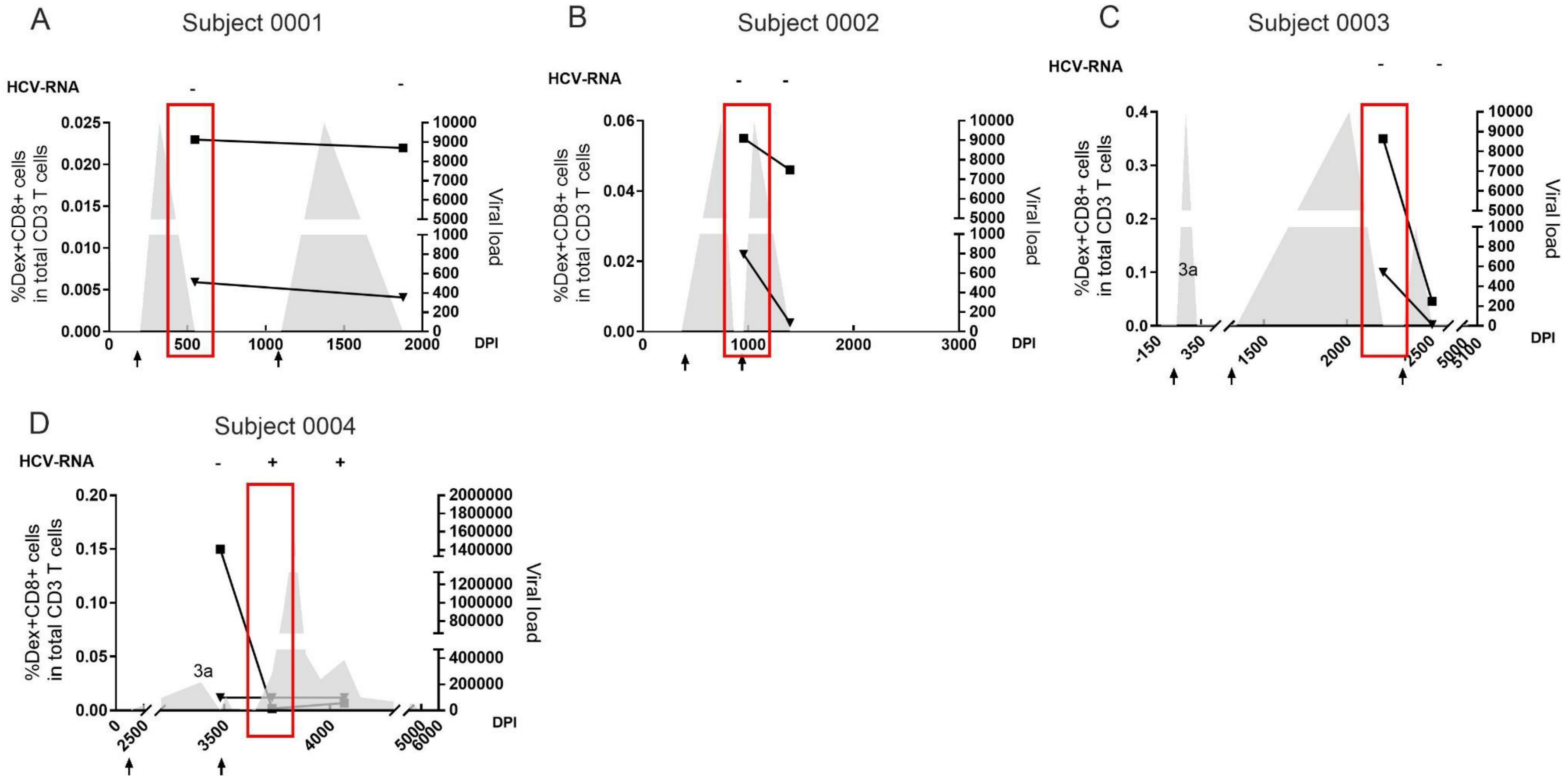

Supplementary Figure 5 Longitudinal analysis of the frequencies of Dex+ CD8 T cells at pre-expansion and post-expansion stage in super-clearers 0001, 0002, and 0003 (A-C), and clearer-chronic subject 0004 (D). *The kinetics of Dex+ CD8 T cell recall responses stimulated with cognate peptide and IL-2/IL-15 as detected by dextramer staining. Inverted triangles represent the Dex+ CD8 frequencies at the pre-expansion stage; the square represents the Dex+ CD8 frequencies at the post-expansion stage. The*

data are derived from independent experiments at individual timepoints from super-clearers (n = 6 samples, 3 donors) and clearer-chronics (n = 3 samples, 1 donor). Grey shaded areas denote the HCV viral load. Each arrow below the x-axis indicates onset of an HCV infection, and DPI represents the days post primary infection. The red boxes indicate the key timepoints prior to re-infection.

.

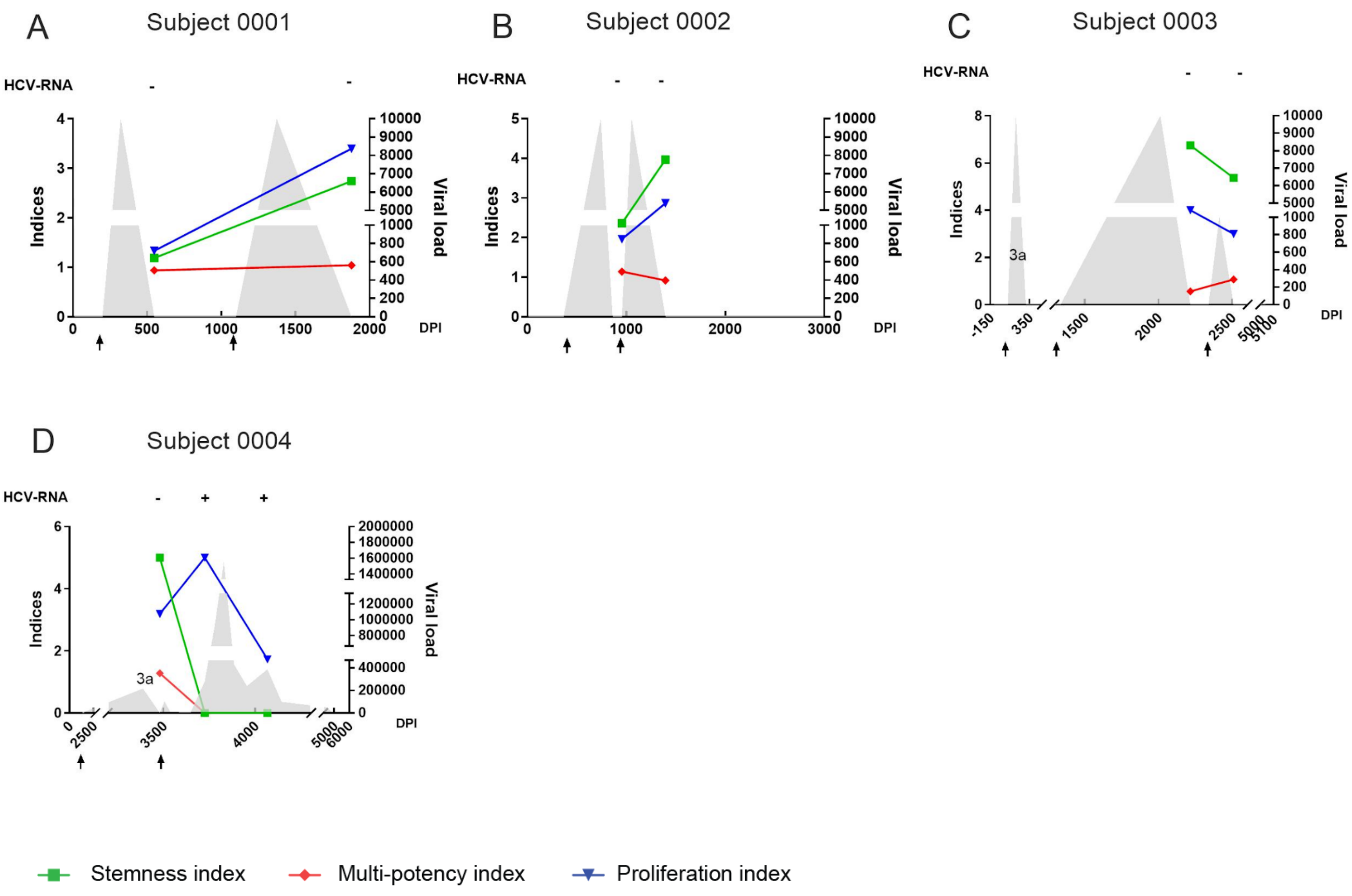
A
Subject 0001
HCV-RNA
-
-
Indices
Viral load
DPI
B
Subject 0002
HCV-RNA
-
-
Indices
Viral load
DPI
C
Subject 0003
HCV-RNA
-
-
3a
Indices
Viral load
DPI
D
Subject 0004
HCV-RNA
-
+
+
3a
Indices
Viral load
DPI
Stemness index
Multi-potency index
Proliferation index

Supplementary Figure 6 Longitudinal analysis of the proliferation index, multi-potency index, and stemness index of Dex+ T_{SCM} after expansion in super-clearers subject 0001, 0002, and 0003 (A-C) and clearer-chronic 0004 (D). *The inverted blue triangle represents the proliferation index of Dex+ T_{SCM}; the red diamond represents the multi-potency index; while the green square represents the stemness index. The grey shaded areas denote the viral load associated with the HCV infection episodes. Each arrow on the x-axis indicates onset of the HCV infection episode, and DPI indicates the days post infection. The data are derived from independent experiments at individual timepoints from super-clearers (n = 6 samples, 3 donors) and clearer-chronics (n = 3 samples, 1 donor)..*

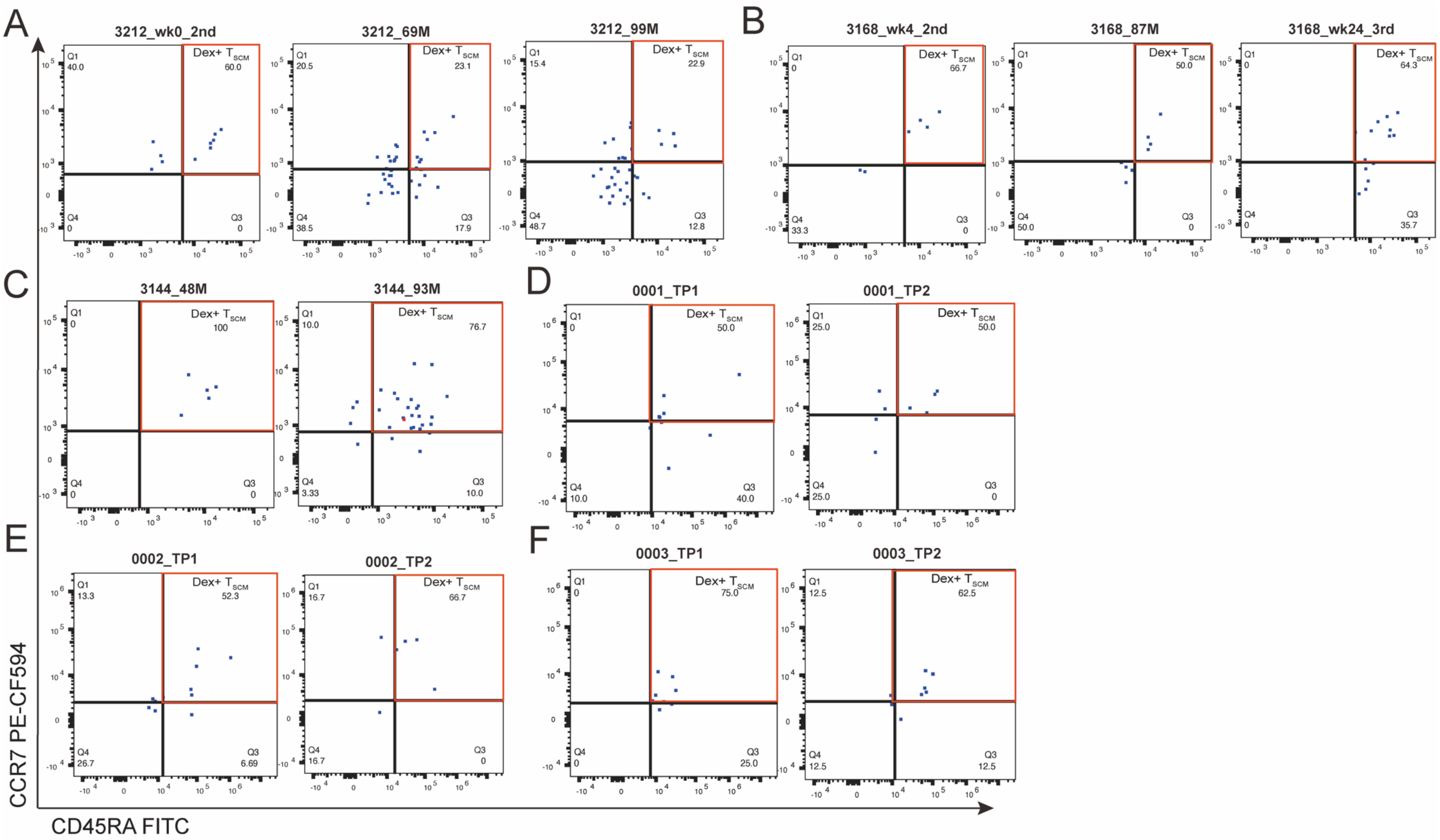
A
3212_wk0_2nd
3212_69M
3212_99M
B
3168_wk4_2nd
3168_87M
3168_wk24_3rd
C
3144_48M
3144_93M
D
0001_TP1
0001_TP2
E
0002_TP1
0002_TP2
F
0003_TP1
0003_TP2
CCR7 PE-CF594
CD45RA FITC

Supplementary Figure 7 Flow cytometry plots showing the frequency of Dex+CTV+ CD8+ T-cell subsets after expansion with cognate peptides and IL-2/IL-15 stimulation. *The red box indicates the Dex+CTV+ T_{SCM} population. The representative gating strategy used to define these subsets is shown in Fig. 4A (bottom panel). Data are derived from independent experiments at individual timepoints from super-clearers (n = 14 samples; 6 donors).*

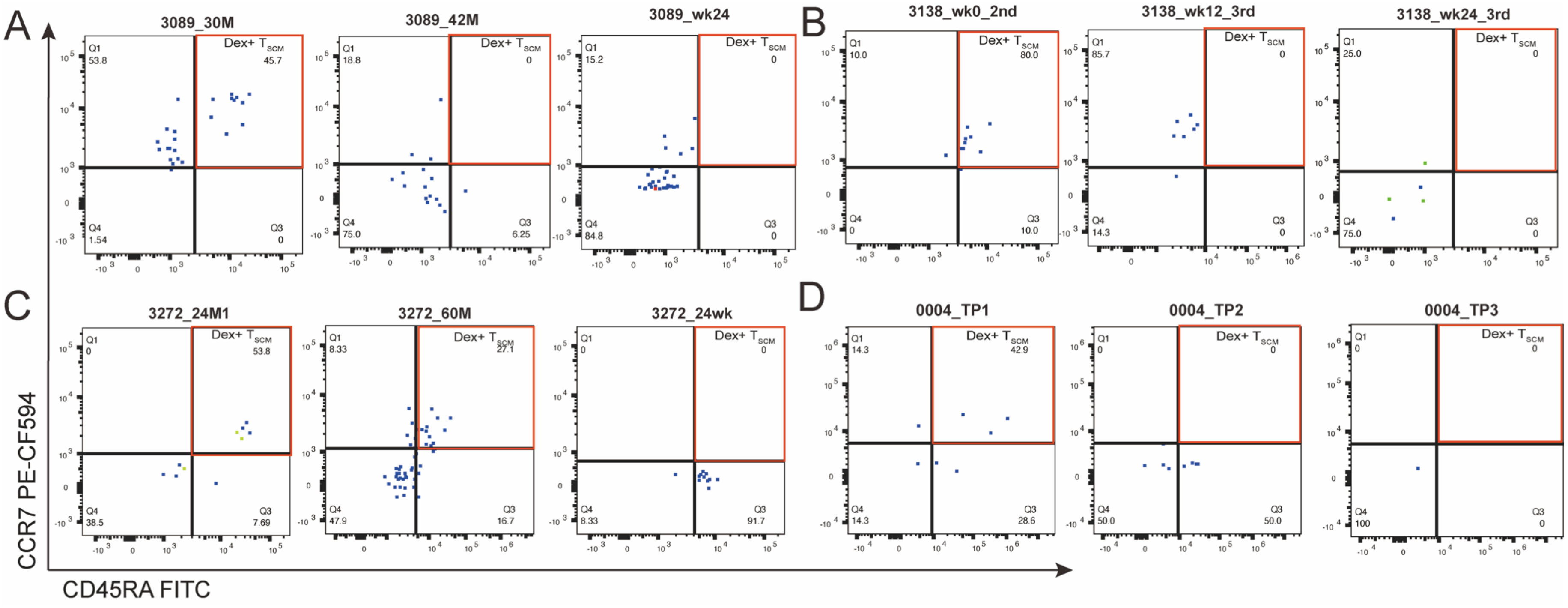

Supplementary Figure 8 Flow cytometry plots showing the frequency of Dex+CTV+ CD8+ T-cell subsets after expansion with cognate peptides and IL-2/IL-15 stimulation. *The red box indicates the Dex+CTV+ T_{SCM} population. The representative gating strategy used to define these subsets is shown in Fig. 4A (bottom panel). Data are derived from independent experiments at individual timepoints from clearer-chronics (n = 12 samples; 4 donors).*

Supplementary Table 1 List of commercial MHC Class I dextramers used for screening of HCV-specific CD8 T cells

HCV protein	Position (aa)	Epitope sequence	HLA restriction
**NS3**	1436-1444	ATDALMTGY	A*0101
**NS3**	1436-1444	ATDALMTGF	A*0101
**NS3**	1073-1081	CINGVCWTV	A*0201
**NS3**	1406-1415	KLVAMGINAV	A*0201
**E2**	610–618	HYPYRLWHY	A*2402
**Core**	51-59	KTSERSQPR	A*0301
**NS3**	1262-1270	LGFGAYMSK	A*0301
**Core**	41-49	GPRLGVRAT	B*0702
**Core**	111-119	DPRRRSRNL	B*0702
**NS3**	1395- 1403	HSKKKCDEL	B*0801
**NS3**	1639-1646	HPVTKYIM	B*0801
**NS3**	1359-1367	HPNIEEVAL	B*3501
**NS3**	1175-1183	HAVGIFRAA	B*3501

Supplementary Table 2 List of U-Load dCODE dextramers for each HLA restriction used for screening of HCV-specific CD8 T cells

HCV protein	Position (aa)	Epitope sequence	HLA restriction
**NS5A**	2224-2233	AELIEANLLW	B*44:02
**NS4B**	1743-1752	AEVIAPAVQT	B*44:02
**NS5B**	2939-2948	AICGKYLFNW	B*44:02
**NS3**	1201-1210	LETTMRSPVF	B*44:02
**Core**	131-140	ADLMGYIPLV	A*02:01
**NS3**	1073-1081	CINGVCWTV	A*02:01
**Core**	132-140	DLMGYIPLV	A*02:01
**NS3**	297-306	KLVAMGINAV	A*02:01
**NS4B**	1807-1816	LLFNILGGWV	A*02:01
**NS3**	1080-1088	TVYHGAGTK	A*03:01
**Core**	51-59	KTSERSQPR	A*03:01
**NS3**	1262-1270	LGFGAYMSK	A*03:01
**Core**	111-119	DPRRRSRNL	B*07:02
**Core**	41-49	GPRLGVRAT	B*07:02

Supplementary Table 3 Commercial and ULoad dCODE MHC Class I dextramers used for each subject.

**Sample**	**HLA restriction (s)**	**Epitope sequence (s) used in each dextramer**
3212	HLA-A*0201	CINGVCWTV
3168	HLA-A*0201	CINGVCWTV
3144	HLA-A*0101	ATDALMTGY
3272	HLA-A*0101	ATDALMTGY
3138	HLA-A*0101	ATDALMTGY
3089	HLA-B*0702	GPRLGVRAT
0001	HLA-A*0201; HLA-B*4402	Pool 1
0002	HLA-B*4402	Pool 2
0003	HLA-A*0201; HLA-B*0702	Pool 3
0004	HLA-A*0201; HLA-B*4402	Pool 4

	**HLA restriction (s)**	**Epitope sequence (s) used in each dextramer**
Pool 1	HLA-A*0201	ADLMGYIPLV
	HLA-A*0201	CINGVCWTV
	HLA-A*0201	DLMGYIPLV
	HLA-A*0201	KLVAMGINAV
	HLA-A*0201	LLFNILGGWV
	HLA-B*4402	AELIEANLLW
	HLA-B*4402	AEVIAPAVQT
	HLA-B*4402	AICGKYLFNW
	HLA-B*4402	LETTMRSPVF

	HLA restriction (s)	Epitope sequence (s) used in each dextramer
Pool 2	HLA-B*4402	AELIEANLLW
	HLA-B*4402	AEVIAPAVQT
	HLA-B*4402	AICGKYLFNW
	HLA-B*4402	LETTMRSPVF

	HLA restriction (s)	Epitope sequence (s) used in each dextramer
Pool 3	HLA-A*0201	ADLMGYIPLV
	HLA-A*0201	CINGVCWTV
	HLA-A*0201	DLMGYIPLV
	HLA-A*0201	KLVAMGINAV
	HLA-A*0201	LLFNILGGWV
	HLA-B*0702	DPRRRSRNL
	HLA-B*0702	GPRLGVRAT
	HLA-B*0702	TVYHGAGTK
	HLA-A*0301	KTSERSQPR
	HLA-A*0301	LGFGAYMSK

	HLA restriction (s)	Epitope sequence (s) used in each dextramer
Pool 4	HLA-A*0201	ADLMGYIPLV
	HLA-A*0201	CINGVCWTV
	HLA-A*0201	DLMGYIPLV
	HLA-A*0201	KLVAMGINAV
	HLA-A*0201	LLFNILGGWV
	HLA-B*4402	AELIEANLLW
	HLA-B*4402	AEVIAPAVQT
	HLA-B*4402	AICGKYLFNW
	HLA-B*4402	LETTMRSPVF

Supplementary Table 4 The percentages of total Dex+ CD8 T cells from super-clearers and clear-chronics at the pre-expansion stage and post-expansion stage.

**Super-clearers**			**clear-chronics**		
sample	pre-expansion	post-expansion	sample	pre-expansion	post-expansion
3212_wk0_2nd	0.21	0.2	3272_24M1	0.220	0.15
3212_69M	0.085	0.24	3272_60M	0.035	0.023
3212_99M	0.15	0.49	3272_24wk	0.037	0.23
3168_wk4_2nd	0.023	0.12	3138_wk0_2nd	0.012	0.022
3168_87M	0.004	0.035	3138_wk24_2nd	0.010	0.005
3168_wk24_3rd	0.009	0.11	3138_wk12_3rd	0.045	0.084
3144_54M	0.001	0.024	3138_wk24_3rd	0.089	0.049
3144_93M	0.2	0.059	3089_30M	0.039	0.58
0001_TP1	0.006	0.023	3089_42M	0.014	0.052
0001_TP2	0.004	0.023	3089_wk24	0.034	0.88
0002_TP1	0.022	0.055	0004_TP1	0.012	0.2
0002_TP2	0.003	0.046	0004_TP2	0.012	0.01
0003_TP1	0.100	0.35	0004_TP3	0.012	0.024
0003_TP2	0.042	0.52			

*TP: timepoints

Supplementary Table 5 The percentages of T_{SCM} among total Dex+ CD8 T cells from super-clearers and clearer-chronics at the pre-expansion stage and post-expansion stage.

**Super-clearers**			**Clearer-chronics**		
sample	pre-expansion	post-expansion	sample	pre-expansion	post-expansion
3212_wk0_2nd	1.6	3.3	3272_24M1	3.54	2.56
3212_69M	20.9	5.2	3272_60M	0.41	1.48
3212_99M	9.8	3.2	3272_24wk	1.23	10.6
3168_wk4_2nd	14.8	29.3	3138_wk0_2nd	13.5	33.3
3168_87M	28.9	93.3	3138_wk24_2nd	5.26	12.5
3168_wk24_3rd	76.2	51.4	3138_wk12_3rd	3.12	0.77
3144_54M	50	88	3138_wk24_3rd	2.04	28.6
3144_93M	4.3	37.9	3089_30M	3.46	2.41
0001_TP1	43.5	11.8	3089_42M	0	0.98
0001_TP2	69.2	15.4	3089_wk24	7.42	0
0002_TP1	53.4	38.2	0004_TP1	61.9	2.73
0002_TP2	20.0	29.4	0004_TP2	68.2	0
0003_TP1	6.3	1.2	0004_TP3	74.1	0
0003_TP2	4.3	0.8			
median	20.45	22.35		3.54	2.41
IQR	41.2	34.35		11.46	9.83

*TP: timepoints

Supplementary Table 6 Alignment of the reference epitopes with the available sequences targeted by Dex+ CD8 T cells in super-clearers and clearer-chronics at primary infection or re-infection with the reference sequences.

HLA type / conserved epitope	Subjects / Genotype	Primary infection	Re-infection
*HLA-A*0201 / NS3-1073*	*3212-1b*	C I NGVCWTV<br>CVNGVCWTV<br>* * * * * * * *	
*HLA-A*0201 / NS3-1073*	*3168-1a*		CINGVCWTV<br>CINGVCWTV<br>* * * * * * * * *
*HLA-A*0101 / NS3-1436*	*3144-3a*	ATDALMTGY<br>ATDALMTGF<br>** ******	
*HLA-A*0101 / NS3-1436*	*3144-1a*		ATDALMTGY<br>ATDALMTGF<br>** ******
*HLA-A*0101 / NS3-1436*	*3272-3a*	ATDALMTGY<br>ATDALMTGF<br>** ******	
*HLA-A*0101 / NS3-1436*	*3138-3aβ*		ATDALMTGY<br>ATDALMTGF<br>** ******
*HLA-B*0702 / Core-41*	*3089-1b/3aα*	GPRLGVRAT<br>GPRLGVRAT<br>*********	
*HLA-B*0702 / Core-41*	*3089-3aβ*		GPRLGVRAT<br>GPRLGVCAT<br>****** **

Supplementary Table 7 MHC-I binding prediction of autologous viral epitope sequences and their reference sequences with IEDB analysis

**allele**	**length**	**peptide**	**IC$_{50}$**	**rank**
**HLA-A*01:01**	9	ATDALMTGY	3.28	0.01
**HLA-A*01:01**	9	ATDALMTGF	235.17	0.32
**HLA-A*02:01**	9	CINGVCWTV	135.48	1.2
**HLA-A*02:01**	9	CVNGVCWTV	268.23	1.9
**HLA-B*07:02**	9	GPRLGVRAT	15.41	0.05
**HLA-B*07:02**	9	GPRLGVCAT	69.08	0.24
